# Transcriptional reprogramming and phenotypic switching associated with the adaptation of *Lactobacillus plantarum* C2 to plant niches

**DOI:** 10.1038/srep27392

**Published:** 2016-06-07

**Authors:** Pasquale Filannino, Raffaella Di Cagno, Carmine Crecchio, Caterina De Virgilio, Maria De Angelis, Marco Gobbetti

**Affiliations:** 1 Department of Plant, Soil and Food Sciences, University of Bari A. Moro, Via Amendola 165/a, 70126 Bari, Italy; 2 Department of Biosciences, Biotechnologies and Biopharmaceutics, University of Bari, Via Orabona, 4, 70126 Bari, Italy

## Abstract

*Lactobacillus plantarum* has been isolated from a large variety of ecological niches, thus highlighting its remarkable environmental adaptability as a generalist. Plant fermentation conditions markedly affect the functional features of *L. plantarum* strains. We investigated the plant niche-specific traits of *L. plantarum* through whole-transcriptome and phenotypic microarray profiles. Carrot (CJ) and pineapple (PJ) juices were chosen as model systems, and MRS broth was used as a control. A set of 3,122 genes was expressed, and 21 to 31% of genes were differentially expressed depending on the plant niche and cell physiological state. *L. plantarum* C2 seemed to specifically respond to plant media conditions. When *L. plantarum* was cultured in CJ, useful pathways were activated, which were aimed to sense the environment, save energy and adopt alternative routes for NAD^+^ regeneration. In PJ the acidic environment caused a transcriptional switching, which was network-linked to an acid tolerance response involving carbohydrate flow, amino acid and protein metabolism, pH homeostasis and membrane fluidity. The most prominent phenotypic dissimilarities observed in cells grown in CJ and PJ were related to carbon and nitrogen metabolism, respectively. Summarising, a snapshot of a carrot and pineapple sensing and adaptive regulation model for *L. plantarum* C2 was proposed.

Microbes in general and bacteria in particular are extremely efficient in filling the niches available in the biosphere^1^. *Lactobacillus plantarum*, a Gram-positive and facultative heterofermentative lactic acid bacteria species, is exemplary in terms of its capacity to adopt successful metabolic strategies. This bacterium has been isolated from a large variety of ecological niches, thus highlighting its remarkable environmental adaptability as a generalist^2^. Metabolic efficiency is among the primary driving forces of bacterial adaptability and consequently of evolution. Bacterial cells adopt high metabolic efficiency strategies, which may result in good fitness^3^. The genetic blueprint of an organism predetermines its ability to adapt to changing environments. The genome of an organism encodes its functional responses and gene regulation mechanisms. The transcription factors and other regulatory components encoded by genomes determine bacterial response patterns.

The genomes of five strains of *L. plantarum* have been sequenced completely or partially^4^,^5^. A comparative analysis has provided detailed insight into the core, variable and accessory genes, as well as gene cassettes, genome synteny, transposable elements, and adaptations on various substrates^6^. An extensive molecular and post-genomics toolbox has been established for *L. plantarum*. This microbe has become one of the model microorganisms used to study lactic acid bacteria^6^. Studies based on genome-wide analysis of gene expression, using *L. plantarum* as a model organism, have been conducted to elucidate strain-specific differences in genome composition^7^,^8^, to validate the use of different techniques for transcription analysis^9^, and to predict parts of the regulatory network^10^ and adaptations to various growth conditions^11^,^12^. *L. plantarum* is often found in plant, dairy, meat, fish, and wheat fermentation and is a natural inhabitant of the gastrointestinal tracts of humans and animals^2^,^13^,^14^,^15^. *L. plantarum* is most abundant in the fermentation of plant-derived raw materials, including several industrial and artisan food and feed fermentations. Accumulating evidence indicates that plant fermentation conditions and storage conditions, as well as the chemical compositions of plant matrices, markedly affect the functional features of *L. plantarum* strains^8^,^16^,^17^. Various metabolomic approaches and multidimensional statistical analyses have recently been used to investigate the metabolic responses of *L. plantarum* strains under different environmental conditions, such as those of vegetable (carrot and tomato) and fruit (pineapple and cherry) media^17^. Owing to its extremely acidic environment, buffering capacity, high concentration of carbohydrates, indigestible nutrients (e.g., fibre), and antinutritional and inhibitory factors (e.g., phenols), pineapple juice has been found to be a highly unfavourable habitat for microbial growth^17^. Almost all tested strains showed specific metabolic responses when grown in pineapple juice. On the contrary, carrot juice does not exert the same metabolic pressure. *L. plantarum* C2 isolated from carrots has been found to show the highest fitness during maintenance of plant materials^17^. The adaptive success of this bacterium in different environmental habitats such as carrot (favourable) and pineapple (unfavourable) juices requires further examination. In fact, the wide colonization of plant environments and the widespread industrial application of *L. plantarum* suggest the need for a better understanding of its genetic responses and metabolism; such knowledge could be used to optimize survival during industrial and downstream processing^8^. Previous transcriptomic studies in *L. plantarum* used de Man, Rogosa and Sharpe (MRS) broth, the standard rich medium for laboratory cultivation^8^. During the cultivation of *L. plantarum* in MRS broth or other laboratory media, this bacterium encounters different conditions from those encountered during plant habitat colonization. To the best of our knowledge, only one transcriptomic study has been performed using *L. plantarum* cells cultivated on hygienic and contaminated carrot juice^18^. The designed macroarray printed on nitrocellulose filters included only 178 selected genes. Recently, specific mechanisms of proteomic adaptation (ca. 80 proteins) has also revealed significant changes in the fermentation profiles of *L. plantarum* strains previously grown in tomato juice compared to cultivation in MRS broth^19^. Despite the above studies and recent progress in unravelling the details of the *L. plantarum* genome, how the bacterium adapts to various plant conditions *via* transcriptional changes remains largely unclear. Our understanding of adaptive strategies cannot be completely drawn from phenotypic responses, which are the final expressions of genomic information. The correlation between complete genotypes (genomes) and complete phenotypes (phenomes) is among the most challenging tasks in biology and has important consequences for theoretical and applied biology such as adaptomics. Phenomics plays a pivotal role in linking genomes and transcriptomes to potential biological functions.

In order to deepen the behaviour of *L. plantrum* during growth and maintenance under plant-like conditions and to identify the specific metabolic pathways, a whole-transcriptome analysis based on customized microarray profiles and a high-throughput phenotypic microarray based on 567 carbon (190) and nitrogen (377) sources were undertaken. These *in vivo* transcriptomes and phenomes were compared to the results for laboratory-cultivated bacteria grown in standard rich medium (MRS).

## Results

### Transcriptional reprogramming during the growth and maintenance of *Lactobacillus plantarum* C2 in plant niches

**Figure 1:**
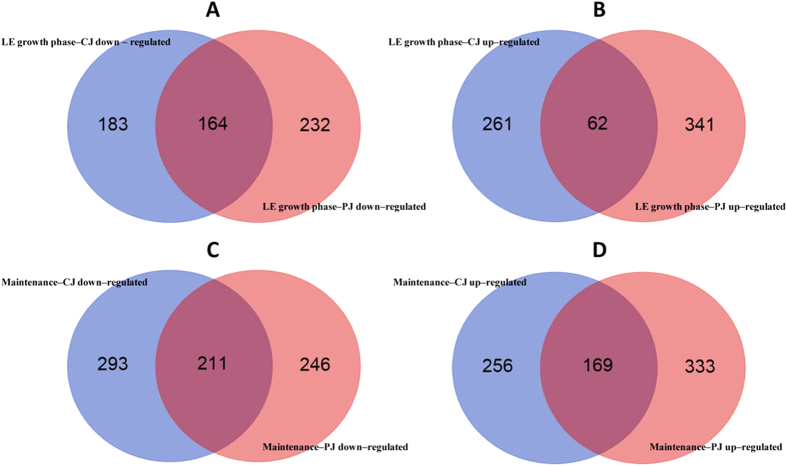
Venn diagrams representing genes that were down- (**A,C**) and up-regulated (**B,D**) (at least two-fold) in *Lactobacillus plantarum* C2 during the late exponential (LE) growth phase (16–18 h at 30 °C) (**A,B**) and during the maintenance period (21 days at 4 °C) (**C,D**) in carrot juice (CJ) and pineapple juice (PJ) compared with MRS medium (FDR adjusted p-value ≤ 0.1 and fold changes ≥ 2 or ≤−2).

**Figure 2:**
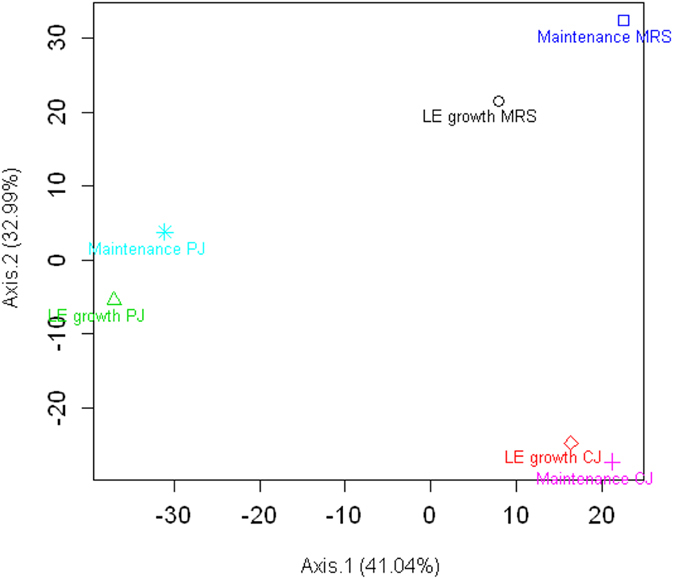
Principal Coordinate Analysis (PCoA) based on transcriptional patterns of *Lactobacillus plantarum* C2 during the late exponential (LE) growth phase (16 or 18 h at 30 °C) and the maintenance period (21 days at 4 °C) in carrot (CJ) and pineapple (PJ) juices and MRS medium. The first two eigenvectors were plotted. Variations explained by the eigenvectors are shown in brackets in the axis labels.

To gain insights into the physiological adaptation of *L. plantarum* C2 to plant (vegetable and fruit) niches, carrot juice (CJ) and pineapple juice (PJ) were chosen as model systems, and the rich medium MRS was used as a control. PJ showed the lowest pH value as well as other intrinsic features (e.g., the highest concentrations of soluble solids, organic acids, and total phenols) that made it the most unfavourable habitat for microbial growth (Table 1). The transcriptomes of C2 in CJ, PJ and MRS were monitored during the late exponential (LE) growth phase and after 21 days of maintenance (see Supplementary Fig. S1). DNA microarrays based on the annotated open reading frames of *L. plantarum* WCFS1 were used. Gene transcription was measured using customized microarray chips consisting of 9,107 probes; two biological replicates were used for each condition (see Dataset S1 in the Supplementary Information). The resulting data exhibited strong cross-chip correlation (R^2^ > 0.95) and good inter-array reproducibility. These data allowed us to define a set of 3,122 genes in *L. plantarum* C2 that were expressed under the experimental conditions of this study (see Supplementary Dataset S1). Global gene expression patterns were visualized by volcano plots (see Supplementary Fig. S2). A comparison of the expression of genes across the two plant niches and the rich medium revealed that C2 exhibited medium-specific transcriptional responses (Figs 1 and 2). A gene was considered to be differentially expressed (DE) in the two conditions (plant substrates *vs* MRS) when its expression level differed at least >2-fold, with an FDR-corrected p-value cut-off of 0.1. We performed pairwise comparisons between the different conditions (plant substrates *vs* MRS). The PJ conditions resulted in a greater number of DE genes during the LE phase of growth compared to the CJ conditions (Fig. 1 and Supplementary Fig. S2, Datasets S2). Maintenance in both plant substrates for 21 days resulted in an increase in the number of DE genes, suggesting that maintenance induced on *L. plantarum* additional adaptive responses (Fig. 1 and Supplementary Dataset S2). The fold-change ratio generally varied between 2- and 10-fold. When the fold change exceeded a factor of 10, the genes were among the least abundant in the data set (see Supplementary Fig. S2 and Dataset S2). The whole-transcriptome results were confirmed by quantitative real-time reverse-transcription PCR (qRT-PCR). It has often been reported that certain quantitative differences in gene expression levels between the two methods could be observed^20^.

**Figure 3:**
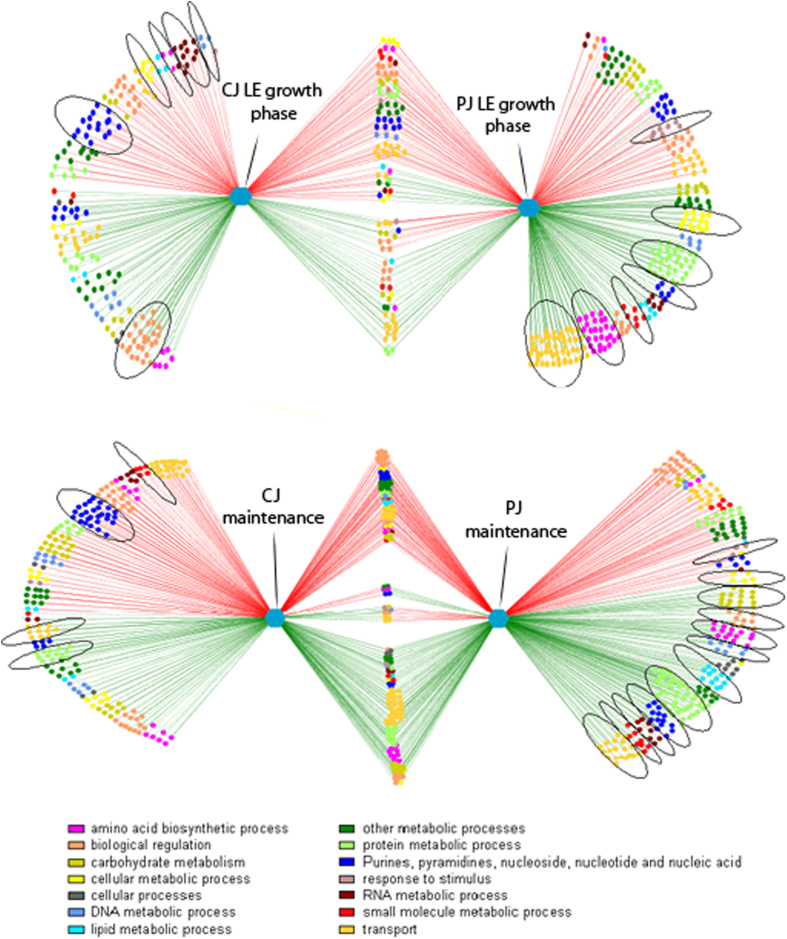
Significantly differentially transcribed genes in *Lactobacillus plantarum* C2 at the late exponential growth phase (16–18 h at 30 °C) (**A**) and during the maintenance period (21 days at 4 °C) (**B**) in carrot juice (left) and pineapple juice (right) compared with MRS medium. The green and red lines indicate up- and down-regulated genes, respectively. The Cytoscape software was used to construct the networks. Each node in the network represents a gene. Edges are coloured according to the up- or down-regulation of the genes; nodes are coloured according to the parent Gene Ontology (GO) term. Significant GO terms with at least 60% of genes having p-values less than 0.05 are circled. Genes from both comparisons (common genes) are not circled, because they were already considered in calculating p-values within individual comparisons.

The visualization of DE genes in networks was based on Gene Ontology (GO) (Fig. 3). GO analyses were obtained by using DAVID (http://david.abcc.ncifcrf.gov/), a web-based tool developed for GO-ranking analysis. Only ontologies with at least 2 genes were considered. Among the biological processes associated with growth in CJ, the largest group of DE genes corresponded to biological regulation (GO:0065007; mainly two-component system genes and transcription regulator genes), purine and pyrimidine nucleotide biosynthesis (GO:0006164 and GO:0006221; 28 down-regulated genes), transport processes (GO:0006810; mainly phosphotransferase system genes and oligopeptide and amino acid transporter genes), and RNA metabolic processes (GO:0016070; mainly down-regulated aminoacyl-tRNA biosynthesis genes). During the maintenance period, protein metabolism and modification processes (GO:0019538; e.g., up-regulated genes encoding ribosomal proteins), and carbohydrate metabolism (GO:0005975) were the most represented biological processes, in addition to transport processes, biological regulation, and purine and pyrimidine nucleotide biosynthesis. When the bacteria were cultivated in PJ, the largest group of up-regulated genes corresponded to transport (mainly ABC transporters and oligopeptide and amino acid transporter genes), protein metabolism and modification processes (mainly genes encoding ribosomal proteins), cellular amino acid biosynthesis (GO:0008652) and cellular metabolic processes (GO:0044237), whereas the largest group of down-regulated genes corresponded to biological regulation (e.g., two-component system genes and transcription regulator genes). Genes related to the response to stimulus (GO:0050896; e.g., universal stress protein) were also significantly down-regulated (p-value < 0.05). Most of the significant alterations in gene expression described above were confirmed during the maintenance period; the only exception was the up-regulation of genes corresponding to lipid metabolic processes (GO:0006629; e.g., fatty acid biosynthesis related genes).

### GO enrichment analysis reveals the roles of common DE genes in plant niches

**Figure 4:**
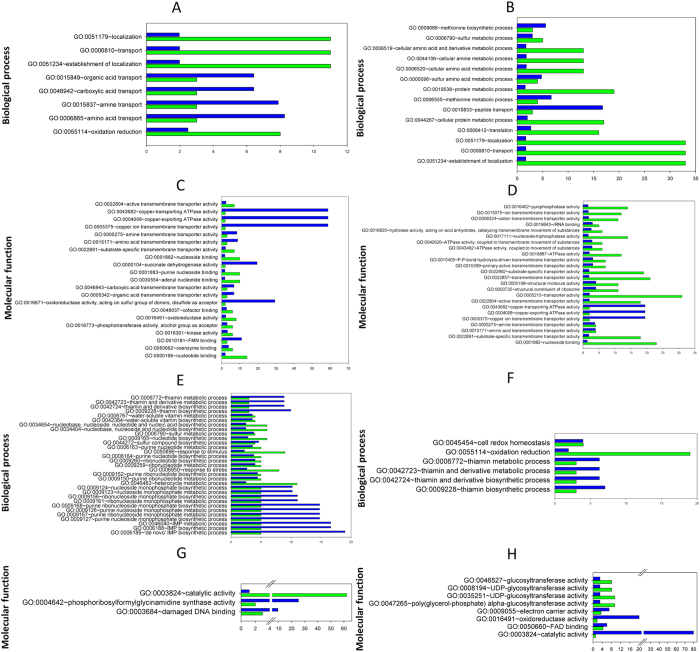
Analysis of functional gene ontology (GO) categories (performed using the DAVID annotation tool) related to the genes differentially expressed in both carrot (CJ) and pineapple (PJ) juices. Only ontologies with at least 2 genes were considered. Fold enrichment (blue bars) and number of genes (green bars) for up-regulated (**A–D**) and down-regulated (**E–H**) biological processes and molecular functions are reported for the late exponential growth phase (16 or 18 h at 30 °C (**A,C,E,G**)) and the maintenance period (21 days at 4 °C (**B,D,F,H**)). Fold enrichment parameter measures the magnitude of enrichment, both for up and down regulated gene groups Analytical modules are provided in the Supplementary Information (Dataset S3).

In each pairwise comparison, a number of DE genes were common to both plant niches during the LE growth phase and during the maintenance period (Figs 1 and 3). During the LE growth phase, most of the common DE genes were down-regulated. The maintenance period resulted in an increase in the number of common DE genes, particularly down-regulated DE genes. To extract the biological meaning behind the common DE genes, a relative ranking of the association of the various GO categories with respect to the gene list was performed using the DAVID tool (Fig. 4). Regardless of the plant substrate used, annotated DE genes represented important biological processes and molecular functions required for the growth and maintenance of *L. plantarum* in plant niches. During the LE growth phase, the most represented biological processes at the transcriptional level (up-regulated genes) were related to transport (referring to the transport of organic acids, amines, amino acids and nucleotides) and oxidation-reduction processes (Fig. 4A and Supplementary Dataset S3). Genes encoding proteins involved in the biosynthesis of vitamins and nucleosides; the metabolism of sulphur, purine and inosine monophosphate; and responses to stimulus and stress (*groEL*, *groES* and *hsp2*) were all among the most significantly down-regulated genes (Fig. 4C and Supplementary Dataset S3). During the maintenance period, genes encoding proteins involved in the metabolism and biosynthesis of cellular amines, amino acids, proteins and sulphur were up-regulated. In contrast, genes encoding proteins involved in cell redox homeostasis and oxidation-reduction processes were among the most common down-regulated genes. In a detailed molecular functional analysis based on GO (Fig. 4B and Supplementary Dataset S3), the most represented categories (up-regulated) were related to binding (of nucleotides, nucleosides, cofactors, coenzymes and rRNA) and to transporter activities (involving amines, amino acids, carboxylic acid, and ions). Genes linked to the structural integrity of ribosomes were up-regulated only during the maintenance period. Groups with other molecular functions, particularly the genes involved in catalytic activity, were down-regulated (Fig. 4D and Supplementary Dataset S3). Fourteen prophage P1 and P2 genes were down-regulated in both CJ and PJ, which included transcriptional regulators (ArpU family) (lp_0656 and lp_2426), genes encoding the large subunit of a terminase (lp_0661 and lp_2466), the replication protein DnaD (lp_2437), and phage-associated lysine (lp_0681) and holin (lp_2399) (Supplementary Table S1). During maintenance, the expression of genes involved in prophage-related functions diversified in opposite ways in the plant juices. In PJ, a number of genes still exhibited reduced expression, whereas in CJ, 18 genes were up-regulated, including genes encoding a major capsid protein (lp_2417), a RusA-like endodeoxyribonuclease (lp_2433), phage Cro/CI family transcriptional regulators (lp_0632 and lp_2448), and a replication protein DnaD domain (lp_2437). Only the gene lp_2397, encoding an extracellular polysaccharide deacetylase, was up-regulated in both plant juices during maintenance. An exhaustive list of all DE genes involved in prophage-related functions is provided in Supplementary Table S1.

### Specific transcriptional changes and related biological functions in *Lactobacillus plantarum* C2 in CJ

**Figure 5:**
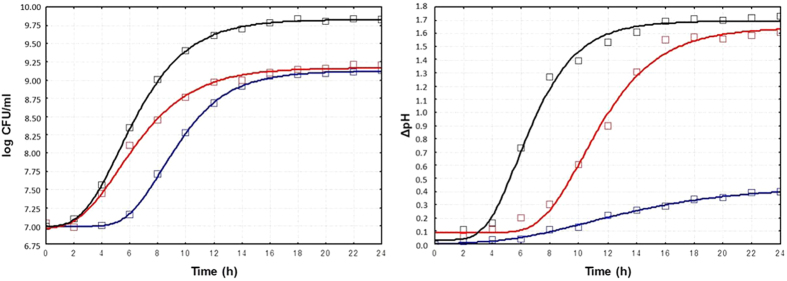
Growth and acidification kinetics of *Lactobacillus plantarum* C2 performed at 30 °C for 24 h in MRS broth (black), carrot juice (red), and pineapple juice (blue).

Microbial growth in CJ was similar to that in the rich MRS medium (9.2 ± 0.05 *vs* 9.8 ± 0.08 log CFU/ml) (Table S2). The values of μmax (0.27 ± 0.02 *vs* 0.39 ± 0.03 cfu ml^−1^ h^−1^) and λ (2.22 ± 0.16 *vs* 2.78 ± 0.22 h), calculated based on the data modelling growth (Fig. 5), were consistent with the final cell densities. Compared to growth in MRS medium, the cell viability of *L. plantarum* C2 slightly (p-value < 0.05) decreased (ca. 0.5 *vs* 0.9 log CFU/ml) during 21 days of maintenance at 4 °C (Table S2). Due to the similar initial pH values of CJ and MRS media (5.81 ± 0.02 *vs* 5.71 ± 0.01), the decrease in the pH of CJ was similar to that observed in MRS medium during LE growth phase (1.55 and 1.69, respectively) and the maintenance period (1.79 and 1.75, respectively) (Table S2). As expected, the concentrations of glucose and fructose decreased markedly (p-value < 0.05) during growth in CJ (14 and 11%, respectively), and the consumption of malic acid was noticeable (p-value < 0.05) during both the LE growth phase (38%) and maintenance (28%) (Table 1 and Supplementary Table S3). Lactic acid was the major fermentation end-product, and acetic acid was detected in trace amounts. Compared to the values prior to fermentation, a marked decrease in total free amino acids (FAA) was found in CJ during the LE growth phase (35%), whereas the concentration of several amino acids increased during maintenance (Table 1 and Supplementary Table S4).

To provide an overview of the specific transcriptional reprogramming in C2 associated with growth and maintenance in CJ, we defined a set of putative KEGG pathways that were significantly enriched and that were associated with enriched genes; the expression of these genes significantly differed in PJ and MRS. The aforementioned DAVID annotation tool was used for pathway analysis. Growth in CJ resulted in a coordinated transcriptional response in C2 during the LE growth phase; this response included the adoption of alternate routes for NAD^+^ regeneration (Supplementary Fig. S3 and Dataset S4). Genes (*lp_3491*, EC:1.3.5.4; *lp_1425*, EC:1.3.5.4; *CitE*, EC:4.1.3.34; and *CitF*, EC:2.8.3.10) associated with the tricarboxylic acid (TCA) cycle were up-regulated (Supplementary Dataset S4). The NAD cofactor was regenerated in C2 *via* the citrate-to-succinate route and part of the TCA cycle to reduce citrate to succinate *via* fumarate. An indirect contribution to NAD cofactor regeneration involved the up-regulation of *ansB* (EC:4.3.1.1), a gene involved in aspartate metabolism. Although not directly involved in the TCA cycle, the product of this gene generates fumarate from aspartate *via* aspartate ammonia-lyase (EC 4.3.1.1). Additionally, we observed the down-regulation of the *gabD* gene of the GABA shunt pathway, which encodes an NAD-consuming succinic semialdehyde (SSA) dehydrogenase (EC 1.2.1.16) that catalyses the oxidation of SSA to succinate. NAD is also salvaged by the significant up-regulation of the *lp_2183* gene (EC:3.6.1.13), which encodes ADP-ribose (ADPR) pyrophosphatase. ADPR is converted to AMP and ribose phosphate (Rib-P) by hydrolases, which may be recycled to generate NAD *via* the formation of PRPP (phosphoribosyl pyrophosphate). Consistently with the whole-transcriptome study, qRT–PCR confirmed that *lp_3491*, *lp_1425*, and *lp_2183* genes were up-regulated, whereas the *gabD* gene was down-regulated (Supplementary Table S5). The growth of C2 in CJ also resulted in the up-regulation of a set of genes involved in environmental sensing and signal transduction; these transcriptional changes were unique to and characteristic of this niche. Genes of two-component systems (TCSs), including the *agr*-like module (*lamA* and *lamC* genes), TCS5 (*hpk5* and *rrp5*), TCS11 (*hpk11* and *rrp11*) and two RR orphans (*lp_0038* of the YycH family and *lp_0039* of the YycI family), were up-regulated. A typical TCS consists of a membrane-associated histidine protein kinase (HPK) and a cytoplasmic response regulator (RR). The former detects specific environmental signals, whereas the latter regulates gene expression. The *agr*-like module *lamA* is known to affect genes involved in activated glucose and galactose production, pyrimidine biosynthesis, and sugar uptake and metabolism^21^. In agreement with this finding, under CJ conditions, *lamA* is apparently involved in global cellular responses, such as the up-regulation of transport (PTS type) for several sugars (such as mannose, fructose and cellobiose, by the related genes *pts9AB*, *fruA* and *pts7C*, respectively), the production of activated glucose (*galE2*, EC:5.1.3.2) and galactose (*galK*, EC 2.7.1.6), and the down-regulation of purine and pyrimidine biosynthesis genes. UDP-galactose is an intermediate in D-galactose metabolism, a known amphibolic metabolic pathway, which affects the expression of various other genes, many of whose functions cannot currently be related to the involved metabolite. The inherent composition of CJ, which may serve as a source of purine and pyrimidine, probably resulted in the down-regulation of genes encoding related metabolic pathways. Specific gene cassettes were up-regulated, such as the csc gene family (*lp_2173*, *lp_2175*, *lp_3073*, *lp_3074*, *lp_3075*, *lp_3412*, *lp_3414*, *lp_3452* and *lp-3453*). These genes encode extracellular proteins involved in the degradation and utilization of plant oligo- or polysaccharides. Several significantly DE genes were assigned to a GO functional category involving translation machinery, such as aminoacyl tRNA synthetase (see Supplementary Fig. S4). We found that many genes (*pheT*, *pheS*, *valS*, *serS2*, *aspS*, *gltX*, *hisS*, *cysS*, *metS* and *ileS*) involved in translation machinery were down-regulated, indicating that growth in CJ slowed translation and reduced the energy needs and cellular physiology in C2. Energy saving was also assured by the down-regulation of genes encoding proteins involved in thiamine metabolism (*xtp1*, EC:3.6.1.66; *thiE*, EC:2.5.1.3; *thiM*, EC:2.7.1.50; and *thiD*, EC:2.7.1.49 2.7.4.7). D-alanine metabolism is linked to a significant acid tolerance response (ATR)^22^. The down-regulation of *dltC1* (EC:6.1.1.13), *alR* (EC:5.1.1.1) and *dltA* (EC:5.1.1.2), which are involved in D-alanine metabolism, may be assumed; growth in CJ does not constitute an acid stress condition for C2. During the maintenance period, the above transcriptomic responses were confirmed in C2 (see Supplementary Fig. S5). However, we identified several DE genes required for maintenance in CJ (see Supplementary Dataset S4). The expression pattern of translation-related genes was affected by the up-regulation of 14 genes encoding 50S (*rplJ*, *rplL*, *rpmB*, and *rplQ*) and 30S (*rpsD*, *rpsC*, *rpsT*, *rpsM*, *rpsO*, *rpsP*, *rpsI*, *rpsJ*, *rpsK*, and *rpsL*) ribosomal proteins (RPs). In addition to protein biosynthesis, RPs also participate in DNA repair, cell death, transcriptional regulation and environmental sensing. The plt locus (*lp_1354a*, *lp_1355* and *lp_1356*) encodes a typical HPK (*pltK*, *lp_1355*) of the HPK10 subfamily and a RR (*pltR* and *lp_1356*). This locus also includes a 58 amino acid double-glycine-type AIP precursor (*pltA*, *lp_1354a*) upstream of *pltK*; this precursor is an additional sensing system activated in C2 during the maintenance period (but not observed during the LE growth phase). However, based on the results from the microarray data, we hypothesized that the salvaging of energy was partly accomplished by down-regulating the biosynthesis of nonessential folate pathway components (*purH*, *dfrA*, *purN*, *fmT*, *glyA* and *thyA*; see Supplementary Dataset S4). During the maintenance period, C2 ensured a supply of sulphur-containing amino acids involved in a variety of cellular functions by up-regulating genes encoding enzymes involved in cysteine and methionine metabolism (*metH*, *thrA2*, *cysE*, *hom1*, *metE*, *hicd2* and *metA*; see Supplementary Dataset S4). Methionine not only is the universal initiator of protein synthesis, but also is involved in active methyl group cycling, polyamine biosynthesis and the trans-sulphuration pathway. Expression of the enzyme ribokinase was highest in MRS broth; however, the expression levels of the phosphoketolase enzyme were similar in all three media. These results suggest that the ribose generated in the phosphoketolase pathway in MRS was most likely used for nucleotide synthesis rather than for energy production. This possibility is supported by the fact that genes for nucleotide synthesis (purines and pyrimidines) were also preferentially expressed in MRS (see Supplementary Figs S3 and S5, Dataset S4, and Table S5).

### *L. plantarum* C2 displays distinct transcriptional adaptations in its core metabolic pathways for growth and maintenance in PJ

The transition to PJ resulted in distinct transcriptional adaptations in C2 in response to an acidic environment typical of fruits. A set of significantly enriched putative KEGG pathways was obtained through DAVID pathway analysis (Supplementary Fig. S6 and Dataset S4). Due to the low initial pH values (3.69 ± 0.02) and high buffering capacity (27.0 ± 0.8 mmol HCl pH^−1^ l^−1^) in PJ, mild lactic acidification occurred during the LE growth phase (3.35 ± 0.02), and the pH remained almost constant during maintenance (3.22 ± 0.01) (Fig. 5 and Table S2). Compared to growth in CJ and MRS medium, PJ induced the longest latency phase (5.74 ± 0.38 h) (Fig. 5). The cell viability during maintenance was 8.35 ± 0.03 log CFU/ml (Table S2). The concentration of glucose, fructose, and sucrose in PJ did not vary significantly (p-value < 0.05), whereas a noticeable consumption (p-value < 0.05) of malic acid was observed in both the LE growth phase (33%) and maintenance (29%) (Table 1 and Supplementary Table S3). The total free amino acids (FAA) decreased (p-value < 0.05) in PJ during the LE growth phase (20%), then markedly increased (p-value < 0.05) during maintenance (157%) (Table 1 and Supplementary Table S4). The acidic environment of PJ altered the transcriptomic profile, which reflected on ATR. The up-regulation of *nhaP2*, a Na^+^/H^+^ antiporter that might be affected by extracellular pH, was observed. We also observed the up-regulation of genes that rerouted pyruvate towards fatty acid biosynthesis. Pyruvate oxidase genes (*pox1, pox3, *and *pox5*; EC:1.2.3.3), which are involved in the conversion of pyruvate to acetyl-coenzyme A (acetyl-CoA), were up-regulated. In addition, the expression of genes encoding pyruvate-consuming enzymes (*ldhL1*, *ldhL2*, and *hicD3*; EC:1.1.1.27) was down-regulated. These results suggest that the altered production of acetyl-CoA was primarily detrimental to lactate production. The downstream utilization of acetyl-CoA appeared to be rerouted towards malonyl-CoA because the transcription of *accA2*, *accD2*, *accC2* (encoding the acetyl-CoA carboxylase subunits alpha and beta and the biotin carboxylase subunit, respectively; EC:6.4.1.2) increased. These molecules are further utilized in fatty acid biosynthesis (*fabH2*, *fabD*, *fabF*, *fabG1*, *fabZ1*, *fabZ2*, *fabI*). The same findings were confirmed by qRT-PCR data (Supplementary Table S5). In PJ, several amino acid metabolism pathways were up-regulated that might play roles in the ATR in C2; these pathways include D-alanine metabolism (*dltD*, *dltC1*, *dltB*, *dltA*, and *dltX* genes), histidine metabolism (10 genes were up-regulated), and aromatic amino acid synthesis (phenylalanine, tyrosine and tryptophan, which are involved in the up-regulation of the shikimate pathway, including the genes *AroA*, *AroB*, *AroC2*, *AroD1*, *AroI*, *AroF*, *trpE*, *trpD*, *trpF*, *trpC*, *trpB*, *trpA*, *tyrA*, and *hisC)*. This pathway may also be involved in redox balancing and NAD regeneration. Several genes encoding branched amino acid transporters, ABC-type oligopeptide transporters (*oppC*, *oppB*, *oppD*, and *oppF*), and two genes involved in intracellular peptidase activity (*pepN*, EC:3.4.11.2; and *pepQ*, EC:3.4.24) were up-regulated. Several additional genes encoding ABC transporters, which release energy from phosphoryl bonds (from ATP) to enable the transport of certain nutrients and minerals, were up-regulated, a result consistent with the qRT-PCR data (Supplementary Table S5). Forty-four genes involved in translation (50S and 30S RPs) were up-regulated during growth in PJ. The removal of protons (H^+^) by F_1_F_0_-ATPase is another example of an ATR mechanism adopted by C2 *via* the up-regulation of the genes *atpA*, *atpB*, *atpC*, *atpD*, *atpE*, *atpF*, *atpG*, and *atpH*. Different genes of the csc cassette (*lp_3458*, *lp_3676*, *lp_3677*, *lp_3678* and *lp_3679*) were up-regulated in C2 grown in PJ. During the LE growth phase in PJ, we observed the down-regulation of genes involved in pyruvate metabolism. This finding suggests that the energy metabolism status is modified. The exposure to high levels of carbohydrates (Table 1 and Supplementary Table S3) probably led to inefficient metabolism and/or catabolic repression, and the bacteria needed to equilibrate the extra- and intra-cellular concentration. Genes encoding phosphotransferase systems (PTS) for acetyl-glucosamine, raffinose, oligosucrose, mannitol and fructose (*pts18CBA*, *rafP*, *pts1BCA*, *pts2cB*, and *fruA*; EC:2.7.1.69) were up-regulated. In contrast, most PTS genes for various carbohydrates (*e.g*., cellobiose and mannose), the second largest transporter classes in *L. plantarum*, were down-regulated. These findings suggest that PEP is allocated to more advantageous pathways for environmental adaptation. During the maintenance period, PEP is converted to pyruvate, as shown by the up-regulation of the *pyK* gene, and competes with PEP-PTS (see Supplementary Fig. S7). Furthermore, pyruvate is converted directly to Acetyl-CoA by two formate C-acetyltransferase enzymes (*pflA*, EC:1.97.1.4; and *pflB*, EC:1.97.1.5) for use in fatty acid biosynthesis. A gene cluster unique to *L. plantarum* involved in sulphur transport and metabolism was highly up-regulated (fold change ranging from 5.3 to 16.9) only in PJ during the LE growth phase of C2. This cluster includes the genes *lp_1378* (EC:2.7.7.4) and *lp_1379* (EC:2.7.1.25), which encode sulphate-converting enzymes (sulphate adenylyltransferase and adenylylsulphate kinase, respectively). The cluster also includes *lp_1380* (EC 3.1.3.7) and *lp_1381* (EC:2.8.2.22), which encode phospho/sulpho-esterase and an extracellular arylsulphate sulpho-transferase, respectively. A sodium/sulphate symport protein (encoded by *lp_1385*) was also up-regulated. Transcriptomic responses observed during the LE growth phase persisted during the maintenance period. A number of up-regulated genes involved in the synthesis of 50S and 30S RPs (elongation factor [EF]-Tu and SecY, RpoA and RpoC,B) were observed. These EFs are primarily responsible for escorting aminoacyl transfer RNAs (tRNAs) to the ribosome. The transcription of genes encoding aminoacyl-tRNA synthetases was up-regulated, most likely due to starvation because of a lack of their cognate amino acids (see Supplementary Fig. S8).

### Phenotypic switching associated with the adaptation of *L. plantarum* C2 to plant niches

**Figure 6:**
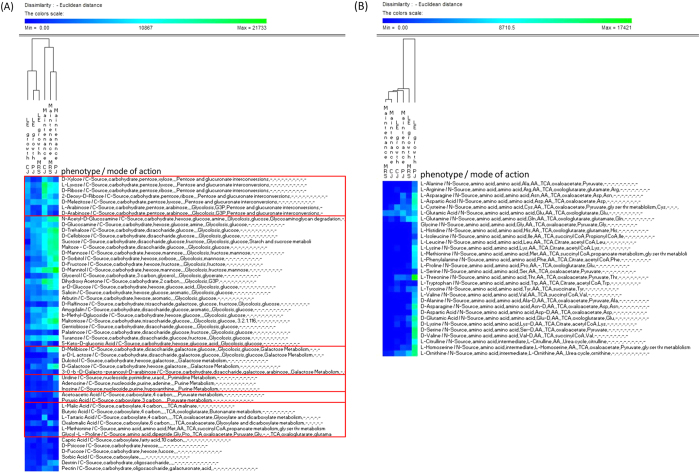
Comparison of *Lactobacillus plantarum* C2 phenotypes during the late exponential growth phase (16 or 18 h at 30 °C) and during the maintenance period (21 days at 4 °C) in MRS, carrot juice (CJ) and pineapple juice (PJ). Each phenotype profile was assayed for growth in the presence of various carbon (**A**) and nitrogen sources (**B**) using OmniLog phenotypic microarrays, as described in the Materials and Methods. Further nitrogen phenotypes and raw data are reported in the Supplementary Information (Dataset S5).

Transcriptional adaptation to environmental changes may lead to phenotypic switching. In a phenotypic screen, we tested whether the expression of genes in C2 varied during growth and maintenance in plant substrates and whether this variability exhibited phenotypic consequences. Cells of C2 in CJ, PJ and MRS were collected during the late exponential (LE) growth phase and after 21 days of maintenance, and used to inoculate the PM plates. Differences in the cell respiration activity of C2 were monitored using the Phenotype MicroArray System (Omnilog), a 96-well plate-based assay, and each well contains unique medium and equivalent tetrazolium dye that develops a purple colour in the reduced form. Cell respiration activity was evaluated through a kinetic response curve bounded by the colour development time-series. The range of phenotypes analysed included the transport, uptake, and catabolism of carbon and nitrogen. Phenotypic assays were performed in duplicate, with high reproducibility (*R*^*2*^ > 0.95 for each metabolite). We show only the most significant (p-value < 0.05) differences in metabolic activities under the experimental conditions used in this study (Fig. 6A,B; Supplementary Dataset S5). During C2 growth and maintenance, both plant substrates induced different phenotypic switching in the utilization of carbon sources. These changes occurred at the levels of pentose and glucuronate interconversion and of glycolysis and galactose metabolism. In CJ, pentose sugars (*e.g*., ribose and arabinose) were highly utilized, whereas in PJ, a major shift towards hexose and hexose derivatives (*e.g*., galactose and mannitol) and oligosaccharides (*e.g*., trehalose, maltotriose, and cellobiose) as sole carbon sources was observed. The increased flux of hexose sugars entering the cell may be a consequence of exposure to high levels of carbohydrates in PJ (Table 1). We also found that PJ stimulated nitrogen metabolism (free amino acids and oligopeptides), particularly during the LE growth phase. Metabolic crosstalk between different metabolism pathways could not be excluded because carbon metabolism is known to regulate nitrogen metabolism in certain bacteria. By simplifying this intricate framework, we were able to describe the phenotypic dissimilarities in C2 among the different plant substrates and MRS medium. Carbon metabolism was most dissimilar in CJ with respect PJ and MRS, and nitrogen metabolism was most dissimilar in PJ compared to CJ and MRS.

## Discussion

**Figure 7:**
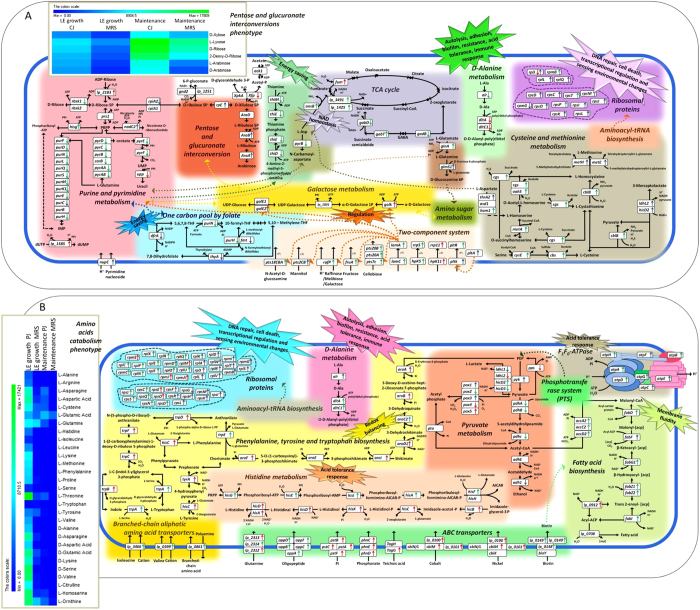
Integrated view of the metabolic pathways affected by transcriptional changes in *Lactobacillus plantarum* C2 during growth and maintenance in carrot (**A**) and pineapple (**B**) juices. Up- and down-regulated genes are indicated by upward and downward pointing arrows, respectively. Red arrows indicate regulation during the late exponential growth phase. Blue arrows indicate regulation during the maintenance period. Green arrows indicate regulation during both growth and maintenance. Correlations between phenotypes (Omnilog microarray) and transcriptional changes are also indicated.

In this study, we generated genome-wide transcriptome and phenotypic microarray profiles of *L. plantarum* in plant niches during growth and maintenance. CJ and PJ were chosen as model systems representative of vegetables (non-acidic environment) and fruits (acidic environment), respectively. Differences in pH values resulted as a significant factor affecting differential gene expression in plant substrates. To our knowledge, this report is the first to present whole-transcriptome and phenome data generated from *L. plantarum* cells cultivated under plant-like conditions. Useful glimpses of this complex biological reality can be gleaned from analysing gene expression under biologically relevant conditions compared to that under rich or artificial media. Whole-genome expression analyses can be combined with phenotypic microarrays to offer a broader picture. The core genome of *L. plantarum* has been proposed to encode traits needed for successful adaptation to and functionality in bacterial niches^2^,^4^. Over 3,000 genes are present in *L. plantarum*, as determined by genome sequencing and comparative genome hybridization studies^2^. We identified a set of 3,122 genes in *L. plantarum* C2 that were expressed under the experimental conditions of this study. The percentage of DE genes ranged from 21 to 31%, depending on the plant niche and cell physiological state. *L. plantarum* C2 appeared to specifically respond to plant signals. Regardless of the plant substrate used, when C2 cells were moved from rich MRS medium to the two plant-like substrates, a common transcriptional response associated with important biological processes was observed during growth (organic acid, amine, amino acid and nucleotide transport and oxidation-reduction processes) and maintenance (cellular amine, amino acid and protein processing, sulphur amino acid metabolism and biosynthetic processes). Although the transition of *L. plantarum* C2 to PJ affected the growth, specifically lengthening the lag phase of growth, the plant substrates did not appear to induce high levels of environmental stress in *L. plantarum* C2, as indicated by the finding that several universal stress-responsive genes were down-regulated compared to cells grown in MRS medium. In other words, universal stress proteins (e.g., heat shock proteins, chaperonins, USP proteins), which usually are stimulated by a large variety of conditions, such as stationary phase, starvation for carbon, nitrogen, phosphate, sulphate and amino acids, and exposure to heat, oxidants, metals, antibiotics and other stimulants^23^, were down-regulated. Similar transcriptomic expression has been found in *Lactococcus lactis* KF147, which was originally isolated from plants, during growth in *Arabidopsis thaliana* leaf tissue lysate^24^. These findings are consistent with the idea that autochthonous lactic acid bacteria from the plant environment are better suited to plant-based fermentation than are allochthonous strains^15^. Although it has been reasoned for decades that metabolically inert prophage DNA would cause a selective disadvantage to the host^25^, the model system applied here clearly suggests that prophage genes are part of the host physiology under specific ecological conditions. Suitable model systems to study phage-host interaction in a relevant ecological context are lacking in the literature, particularly for plant ecosystems. The data provided for such complex genome interactions may indicate a substantial challenge in assessing the role of prophages for the phenotype of *L. plantarum* under plant-like conditions. Considering the adaptation responses observed in this study, we have proposed a carrot and pineapple sensing and adaptive regulation model for *L. plantarum* C2, which is shown in Fig. 7. Alterations at the transcript level are imprinted in C2 during both the LE growth phase and the maintenance period in the carrot model system. Functional pathways are activated to sense the environment, and other core pathways are modulated to save energy and to adopt alternate routes for NAD^+^ cofactor regeneration (Fig. 7A). The citrate-to-succinate route of the TCA cycle was followed to regenerate this cofactor^26^. During fermentation in heterofermentative lactic acid bacteria, citrate metabolism may contribute to energy production by providing a major alternate pathway for NAD^+^ regeneration^4^,^26^. Aspartate metabolism generates fumarate *via* ammonia-lyase and GABA shunt pathway down-regulation, further salvaging NAD^+^. This finding suggests that *L. plantarum*, like other lactobacilli, has a functional citrate metabolism pathway that may lead to succinate production^27^. The GABA shunt is energetically less efficient than the direct oxidation of 2-ketoglutarate to succinate by the TCA cycle. Therefore, visualizing the preferential adoption of this shunt pathway under normal conditions is difficult. The proposed physiological role of ADP-ribose as an anti-repressor of NAD^+^ synthesis is based on the assumption that the cell may interpret the accumulation of ADPR as a signal to replenish the NAD^+^ cofactor pool. This assumption is reasonable, because the only source of ADPR in the cell is the consumption of NAD^+^ through direct enzymatic hydrolysis^28^. The ribose generated in the phosphoketolase pathway in MRS is most likely used for nucleotide synthesis rather than for energy production. This possibility is supported by the fact that the genes for nucleotide synthesis (purines and pyrimidines) are also preferentially expressed in MRS medium. In contrast, in CJ, ribose is utilized primarily in energy production. The preference for pentose sugars in C2 is also observed in phenotypic switching associated with the carrot niche. The signals and/or conditions in the carrot environment seem to stimulate three TCSs in C2. Bacteria generally sense and respond to environmental changes through TCSs^21^,^29^, which are among the most important mechanisms for external environmental sensing and signal transduction^30^,^31^. TCSs are involved in controlling a wide variety of physiological processes that correspond to the regulation of carbohydrate transport under the carrot conditions. During maintenance, the activation of the *pltAKR* operon probably results in the putative quorum sensing control of cell density in C2^29^. Several csc gene cassettes were up-regulated during both the LE growth phase and the maintenance period. The *L. plantarum* WFCS1 chromosome encodes over 200 putative extra-cellular proteins, most of which should be displayed at the cell surface^32^,^33^,^34^. Some of these extracellular proteins are encoded in specific csc gene cassettes^32^, and their primary occurrence in plant-associated Gram-positive bacteria suggests a possible role in the degradation and utilization of plant oligo- or polysaccharides^6^. To save energy, transcriptional reprogramming in C2 most likely involves the down-regulation of nonessential biosynthesis pathways (*e.g*., aminoacyl tRNA synthetase and thiamine metabolism). During growth and maintenance in CJ, *L. plantarum* C2 seemed to prefer to invest energy in other metabolic processes. Saving energy is a metabolic strategy adopted by bacteria to counter environmental conditions^35^. Reduced activity of the electron transport system accounted for a reduction in available ATP, slowing growth in *Staphylococcus aureus* SCVs, which resulted in the down-regulation of the nonessential thiamine metabolism^36^. Similarly, the purine and pyrimidine biosynthesis pathway was down-regulated, suggesting that CJ could provide a sufficient amount of nucleotides; thus, no synthesis was necessary^37^. Another example of energy conservation is the down-regulation of folate biosynthesis during the maintenance period. A decrease in the biosynthesis of folate, a precursor of purine, was most likely linked to the down-regulation of metabolism^38^. Transcriptomic profiling provided evidence that *L. plantarum* responds to prolonged maintenance in CJ by altering its cytoplasmic proteins to maintain cellular homeostasis and hence to facilitate survival. RPs are typically associated with protein synthesis; however, some ribosomes may be secreted to the cell surface or into the external environment as a defence mechanism in response to changing environmental conditions such as those in CJ during the maintenance period^39^. The supply of the sulphur-containing amino acids cysteine and methionine, which are involved in a variety of cellular functions, was recovered during the maintenance period. Methionine is the universal N-terminal amino acid of proteins, and its derivative S-adenosylmethionine is utilized in a variety of methyltransferase reactions. These roles suggest the importance of methionine in cellular metabolism^40^. When PJ was used as a model system, specific transcriptional responses and metabolic pathways were completely modified in C2 cells (Fig. 7B). The metabolic changes that emerged were primarily the result of coordinated ATR. Acid tolerance has been described in numerous lactic acid bacteria such as *Lactococcus lactis* MG1363, *Lactobacillus reuteri* ATCC 23272, *L. bulgaricus* ATCC 11842, *Lactobacillus casei* ATCC 334 and *Lactobacillus rhamnosus* GG^41^. According to previous studies, the mechanisms that underlie ATR are species dependent. The following is a description of the factors involved in ATR in *L. plantarum* under PJ conditions. The up-regulation of the Na^+^/H^+^ antiporter NapA3 is associated with cell wall transport, which might affect survival in acidic environments *via* pH homeostasis^42^. The rerouting of the pyruvate metabolism to favour fatty acid biosynthesis affects membrane fluidity and enhances acid stress resistance^41^,^43^. Membrane lipids have been associated with diverse roles, including respiration, metabolism, protein transport, and peptidoglycan wall synthesis, as well as the initiation of chromosome replication and cell division^44^. The integrity and maintenance of plasma membrane function is vital to cell survival. Alterations in fatty acid composition and membrane fluidity may serve as mechanisms for adapting to changing environments^44^. We postulate that the low pH of plant environments disturbs the cellular balance of free amino acids, which might repress cell growth. Similar findings in *Lactobacillus reuteri* have been reported by Wall *et al*.^45^. The activation of the *dltC* gene, which is involved in D-alanine metabolism, histidine metabolism, and aromatic amino acid synthesis, which is involved in the up-regulation of the shikimate pathway, might play a role in ATR^17^,^22^. Tryptophan biosynthesis is a biologically expensive and complicated process because the products of four other pathways are essential carbon or nitrogen contributors^46^. The precursor chorismate is also the precursor of several other metabolites (*e.g*., *p*-aminobenzoic acid). In addition, glutamine, phosphoribosylpyrophosphate, and L-serine contribute nitrogen and/or carbon during tryptophan formation. Thus, each organism with tryptophan-synthesizing capabilities must have adopted appropriate regulatory strategies to ensure sufficient levels of chorismate. The proteolytic roles of intracellular peptidases as well as of amino acid decarboxylases in adapting to acidic environments have been observed in other lactic acid bacteria^41^,^47^,^48^. Previous studies have indicated that intracellular amino acids might become limiting when cells overcome acidic environments, presumably because of the reduced efficiency of amino acid ABC transporters under acid stress^41^,^49^. This finding is consistent with the lower consumption of FAA in PJ compared to the other media during the LE growth phase. A decrease in the intracellular concentration of amino acids may trigger the cell to obtain amino acids by other means, *e.g*., through the overexpression of peptidases or proteins involved in peptide transport. Branched amino acid transporters and ABC-type oligopeptide transporters were activated under PJ conditions. The clear stimulation of nitrogen metabolism through phenotypic switching was associated with the pineapple niche. Two EFs (EF-Tu and EF-G) are primarily responsible for escorting aminoacyl transfer RNAs (tRNAs) to the ribosome^41^. In addition to their role in translation, these proteins perform a chaperone-like function in protein folding^50^. The interaction between the ribosome and the complex formed between EF-Tu and amino-acyl-tRNA plays a key role in controlling translational accuracy^41^. As in certain lactic acid bacteria species, *L. plantarum* C2 maintains its intracellular pH homeostasis via F_1_F_0_-ATPase under acid stress conditions^41^. Cells experience fluxes in metabolism and energy when adapting to acidic environments, thus suggesting that their energy metabolism status is modified^47^. ABC transporters and phosphotransferase systems, the two largest transporter classes in *L. plantarum* WCFS1, were activated. These transporters employ a sequential mechanism involving several protein-protein interactions to free the energy from phosphoryl bonds (ABC transporters and phosphotransferases hydrolyse ATP and phosphoenolpyruvate, respectively), enabling the transport of certain nutrients (carbohydrates in the case of PTSs) or other molecules across cell membranes^51^. A gene cluster unique to *L. plantarum* was highly up-regulated; genes in this cluster are involved in sulphur transport and metabolism. We speculate that these sulphate-converting enzymes, together with a phospho/sulpho-esterase and an extracellular arylsulphate sulpho-transferase, might be involved in the utilization of sulphate from sulphated polysaccharides found in plants^2^. An intricate phenotypic framework highlights the phenotypic dissimilarity observed in C2 across the different plant substrates and in laboratory culture media. These findings support the transcriptomic data and reveal the cellular mechanisms underlying the adaptation to plant niches. Linking the phenotypes to high-throughput molecular biology data generated by omics technologies allowed us to uncover bacterial phenotypes related to plant-based transcriptomic switching.

The ability to ferment plant substrates is related to the capacity of a bacterium to rapidly adapt and use the available nutrients for growth. The importance of this process, especially for the innovative fermented food industry, has stimulated extensive research. Together, the results presented in this study support the conclusion that *L. plantarum* exhibits high levels of environmental niche specificity to support its growth and survival in different plant-associated habitats. The model system applied here and the reconstruction of the regulatory network will help to elucidate the processes that underlie specific *in situ* behaviour, *e.g*., during food fermentation processes. The carrot substrate influences the behaviour of *L. plantarum* and, in turn, its environmental adaptation and phenotype. We conclude that the strain senses the plant stimulus and adjusts its carbohydrate metabolism, which could increase the strain’s capacity to compete. The chemical composition and acid conditions of the pineapple substrate caused the switching of the bacterial metabolism towards pathways involving the metabolism and catabolism of amino acids, thus modifying the overall plant nutritional and sensory features. Consequently, the combined reconstructed networks could be used to rationalize the discovery of targets for optimizing culture performance and for improving strain robustness, as well as to improve understanding of how lactic acid bacteria transform raw starting materials into economically valuable food, feed, and industrial products.

## Materials and Methods

### Preparation of media

CJ and PJ media were chosen as model systems representative of plant ecosystems (vegetables and fruits, respectively). Juice media were prepared as described by Filannino *et al*.^17^. Briefly, carrot or pineapple was homogenized, centrifuged (10,000 × *g* for 20 min at 4 °C), heat treated (121 °C for 10 min), filtered onto a Whatman apparatus (Polycarp 75 SPF; Whatman International, Maidstone, England), and sterilized by filtration on 0.22 μm membrane filters (Millipore). Rich MRS medium (Oxoid) was used as the control for optimal growth.

### Strain and growth conditions

*Lactobacillus plantarum* C2 obtained from the Culture Collection of the Department of Soil, Plant and Food Science of the University of Bari Aldo Moro (Bari, Italy) was used in this study. *L. plantarum* C2 was isolated previously from carrots^52^. Cultures were maintained as stocks in 15% (vol/vol) glycerol at −80 °C. Culture inocula under the conditions investigated in this study were prepared by harvesting cells during the LE growth phase (ca. 15 h) in MRS broth. The cells were washed twice in 50 mM sterile potassium phosphate buffer (pH 7.0). The initial cell number used to inoculate culture media was ca. 10^7^ CFU/ml. Incubation was performed at 30 °C for 24 h; further maintenance was continued for 21 days at 4 °C. Cell enumeration during growth and after maintenance was conducted by plating onto MRS agar. Growth kinetics were determined and modelled according to the Gompertz equation as modified by Zwietering *et al*.^53^: *y* = *k* + *A* exp{−exp[(*μ*_max_ or *V*_max_ *e*/*A*)(*λ* − *t*) + 1]}, where *k* is the initial level of the dependent variable to be modeled (log CFU/ml or pH units), *A* is the difference in log CFU/ml or pH units (ΔpH) between inoculation and the stationary phase, μ_max_ and *V*_max_ are the maximum growth rate (expressed as log CFU/ml/h) and the maximum acidification rate (expressed as pH/h), respectively, *λ* is the length of the latency phase expressed in hours, and *t* is the time. Fitting procedures and parametric estimations were carried out using the “non-linear estimation” module and the option “user-specified regression, custom loss function” provided by Statistica 7.0 for Windows. The estimators of *A*, *μ*_*max*_, *V*_*max*_, and *λ* were approximated by the application of the algorithm quasi-Newton (Statistica 7.0 for Windows).

Due to the different chemical compositions of the CJ and PJ media and based on growth kinetic data, the LE growth phase of *L. plantarum* C2 was reached after ca. 16 and ca. 18 h, respectively (Fig. 5). Samples were analysed at the LE growth phase in MRS broth, CJ and PJ and after maintenance. Biologically independent duplicates were performed for each condition.

### Physico-chemical analysis

Organic acids and carbohydrates were determined through high-performance liquid chromatography (HPLC) analysis using the Äkta Purifier System (GE Healthcare), which was equipped with an Aminex HPX-87H column (ion exclusion; Bio-Rad) and a UV detector operating at 210 nm or with a Spherisorb column (Waters, Milford, MA, USA) and a PerkinElmer 200a refractive index detector (PerkinElmer, Waltham, MA, USA), as described by Filannino *et al*.^17^. Total and individual free amino acids (FAA) were analysed with a Biochrom 30 series amino acid analyser (Biochrom Ltd., Cambridge Science Park, England)^17^. Total titratable acidity (TTA), soluble solids, total phenol compounds, and buffering capacity were determined as described by Filannino *et al*.^17^. For buffering capacity assay, one-hundred milliliters of each medium was titrated with 1N HCl. The values were expressed as the amount of HCl (mmol) needed to drop 1 pH unit per unit volume (1 liter). Analyses were performed in duplicate with three biological replicates for each growth condition.

### Whole-transcriptome gene expression data

Whole-transcriptome analysis based on customized microarray profiles was used to determine the altered transcription patterns in *L. plantarum* C2. RNA extraction was performed using an RNeasy Mini Kit (Qiagen) with DNase treatment. RNA concentrations and purities were determined at an optical density ratio of 260/280 using a NanoDrop ND-1000 spectrophotometer (NanoDrop Technologies). The integrity of the total RNA samples was verified using an Agilent 2100 Bioanalyzer and an RNA 6000 Nano LabChip (Agilent Technologies). The samples for gene expression analysis were labelled using an Agilent Quick-Amp Labeling Kit (p/n5190–0442). Five-hundred nanograms of each total RNA sample was reverse transcribed at 40 °C using a WT primer (Low Input Quick Amp WT Labeling kit 5190–2943, Agilent Technologies) with a T7 polymerase promoter and converted to double-stranded cDNA. Synthesized double-stranded cDNA was used as the template for cRNA (amino allyl modified) generation. The cRNA was generated by *in vitro* transcription; Cy3 CTP dye (Agilent Technologies) was incorporated during this step^54^. Labelled cRNA was purified using Qiagen RNeasy columns (Qiagen). Quality, yield and specific activity were assessed using a Nanodrop ND-1000. In total, 2000 ng of labelled cRNA sample was fragmented at 60 °C and hybridized onto a custom-designed *Lactobacillus*_GXP_8 × 15 k (AMADID No: 067475) array. Labelled cRNA fragmentation and hybridization were performed using a Gene Expression Hybridization Kit (Agilent Technologies, *in Situ* Hybridization Kit, Part Number 5190–0404). Hybridization was performed for 16 h in Agilent Surehyb Chambers at 65 °C. The hybridized slides were washed using Agilent Gene Expression wash buffers (Agilent Technologies, Part Number 5188–5327) and scanned using an Agilent Microarray Scanner (Agilent Technologies, Part Number G2600D) at 5 micron resolution. Data extraction from the images was performed using Feature Extraction software v11.5 (Agilent).

### Microarray data analysis tools

Text files (.txt format) obtained from the Feature Extraction software were used for analysis. Data from the “gMedianSignal” column (calculated from the intensities of all inlier pixels representing the feature, after outlier pixel rejection) were taken as the foreground intensity, and data from the “gBGMedianSignal” column (median local background signal (local to corresponding feature) computed per channel (inlier pixels)) were taken as the background intensity. The data were background corrected and quantile normalized using the functions in the *limma* package for R. If a gene had more than one probe, the average intensity value of all the probes was used to represent the gene. Pair-wise correlations between the samples were analysed using Pearson’s correlation coefficient. Normalized expression data were used to identify DE genes across the comparison of interest. Gene-wise models were built using all samples, and contrasts were defined for each comparison of interest. The linear modelling approach in the *limma* library for R was used to build models and to define comparisons of interest. A Bayesian-adjusted t-statistic was used to identify DE genes. Because the analysis involved a large number of tests, multiple testing corrections were performed using Benjamini and Hochberg’s FDR. Genes with FDR-adjusted p-values ≤ 0.1 were statistically significant; genes with fold changes ≥ 2 or ≤−2 were considered to be up- or down-regulated, respectively.

### Gene ontology and metabolic pathways

The visualization of DE genes predicted to be significant (see above) were used to draw networks based on GO (biological processes). The Cytoscape software suite (version 3.2.0) was used to generate networks^55^. Under the biological process GO analysis, only genes that were significantly up- or down-regulated were considered for network construction. Gene ontologies were obtained using a DAVID analysis (http://david.abcc.ncifcrf.gov/), and only ontologies with at least 2 genes were considered. For each GO term, the parent GO term was obtained using the QuickGO tool. Each gene was associated with its parent term based on its description. In cases in which a gene had multiple GO terms, the minimum p-values of all terms were assigned to that particular gene. Each gene in the network was represented by a single node. The colouring of the edges was based on the up- or down-regulation of the gene; the colouring of nodes was based on the parent GO term. Significant GO terms are circled; at least 60% of genes had p-values less than 0.05. Genes from both comparisons (common genes) are not circled because they were already considered to have p-values within individual comparisons. Up- and down-regulated genes encoding enzymes are depicted in their respective pathways. The aforementioned DAVID annotation tool was used for pathway analysis. Only significantly up- and down-regulated pathways were considered for generating pathway images. Pathways showing good interconnectivity (based on common substrates) were integrated. KGML files for the above-mentioned pathways were downloaded from the KEGG Pathway database using the organism code “lpl”^56^,^57^,^58^ (http://www.kegg.jp/ or http://www.genome.jp/kegg). KGML files were edited using KGML-ED Pathway Editor v1.9. The nodes for genes encoding enzymes are coloured lime green to represent up-regulated genes and red to represent down-regulated genes, on the basis of pathway mapping. Other nodes for enzymes are represented in pale green and substrates in white. Multiple up- and down-regulated genes are separated by semicolons.

### Validation of microarray data by qRT-PCR

The microarray results were validated by quantitative real-time reverse transcription-PCR (qRT-PCR). Twenty-six genes were chosen among the differentially expressed genes in CJ and PJ (Table S6) for data confirmation. RNA extraction was performed on biologically independent duplicates. Total RNA was obtained using a Qiagen RNeasy^®^ Mini Kit (including the RNase Free DNase set) as described by the manufacturer. RNA concentration was determined by spectrophotometric measurements at 260, 280, and 230 nm using a NanoDrop^®^ ND 1000 spectrophotometer (ThermoFisher Scientific Inc., MI., Italy). The cDNA was synthesized from 1 μg of total RNA using SuperScript^®^ VILOTM, as described by the manufacturer (Invitrogen). The cDNAs were diluted 100 times for real-time PCR experiments. To quantify gene expression in real time, specific oligonucleotide primer pairs were designed against target sequences by NCBI and constructed using the Blast (http://www.ncbi.nlm.nih.gov/tools/primer-blast/) and primer3 (http://frodo.wi.mit.edu/) software. Primers were designed to have nearly identical annealing temperatures. All pairs of primers were tested in real-time PCR experiments. The real-time PCR was performed with the QuantStudio 7 Flex System (Applied Biosystems by Life Technologies) using Itaq Universal Sybr Green Supermix (BioRad) according to the manufacturer’s instructions. Thermal cycling conditions included an initial heat-denaturing step at 95 °C for 15 sec, followed by 40 cycles at 95 °C for 30 sec and 60 °C for 1 min. After amplification, the melting curves of the PCR products were determined from 60 to 95 °C to ascertain the specificity of the amplification. All samples were run in triplicate. RecA and 16S rRNA genes were used as the endogenous controls. For all PCRs, the cycle threshold values were processed by the 2^−ΔΔCT^ method^59^. Quantitative real-time PCR data were analysed through the DART-PCR spreadsheet in Excel version 1.0 using raw fluorescence data. This method of analysing real-time PCR converts raw fluorescence data into R0 values according to the theory that fluorescence is proportional to DNA concentration. This approach allows the automatic calculation of amplification kinetics as well as subsequent calculations for the relative quantification and calculation of assay variability, giving a final estimate of the efficiency of amplification of the primer pairs used in real-time reactions^60^. qRT-PCR data were compared with the whole-transcriptome study (Table S5). A high similarity was found between patterns of fold-change levels of differentially expressed genes detected through both methods, indicating that the biological conclusions drawn from the transcriptome data were reliable.

### Phenotypic microarray analysis

Differences in phenotype under various growth conditions were monitored using OmniLog Phenotype MicroArray (PM) Technology (Biolog). PM plates (Biolog) containing 190 carbon sources (PM1 and PM2) and 377 nitrogen sources, including free amino acids and peptides (PM3, PM6, PM7, and PM8), were used. Phenotypic microarray analyses were performed with two biological replicates for each growth condition in accordance with the manufacturer’s instructions. Cells of C2 in CJ, PJ and MRS were collected during the late exponential (LE) growth phase and after 21 days of maintenance. Cells were washed in 50 mM sterile potassium phosphate buffer (pH 7.0), diluted (to achieve 65% transmittance) in inoculating fluid (Biolog), and used to inoculate the PM plates. One-hundred μl of cell suspension were added per each well. Plates were incubated for 24 h (PM1 and PM2) or four days (PM3, PM6, PM7, and PM8) at 33 °C in an OmniLog automated incubator/reader (Biolog). During incubation, reduction of tetrazolium dye by respiring cells was measured in each well every15 min by the OmniLog system. Cellular respiration activity was evaluated from the area of a region bounded by a colour development time-series. The results were analysed using the Omnilog PM software (Biolog) according to the manufacturer’s instructions. Phenotypic assays were performed in duplicate with high reproducibility (*R*^*2*^ > 0.95 for each metabolite). We show only the most significant (p-value < 0.05) differences in metabolic activities under the experimental conditions of this study.

### Statistical analysis

Data from growth kinetics, chemical and physical analysis of the culture media, qRT-PCR, and phenotypic microarray analysis (at least two replicates) were subjected to one-way analysis of variance (ANOVA), and pairwise comparison of the treatment means was performed by Tukey’s procedure at a p-value < 0.05, using the statistical software Statistica 7.0 for Windows. Principal Coordinate Analysis (PCoA) was used to assess whether the transcriptional patterns of *L. plantarum* C2 were distinct from each other based on the growth media and conditions. Euclidean distances were calculated using the *dist* function available in the *base* package of R. The average expression values of replicates were taken to represent the condition. PcoA was performed using the *pcoa* function in the *ape* package of R. The first two eigenvectors were plotted as shown in Fig. 2. Variations explained by the eigenvectors are shown in brackets in the axis labels.

## Additional Information

**Accession codes**: The data from the microarray experiments described here are available at the Gene Expression Omnibus (GEO [http://www.ncbi.nlm.nih.gov/geo/]) under accession number GSE68188.

**How to cite this article**: Filannino, P. *et al*. Transcriptional reprogramming and phenotypic switching associated with the adaptation of *Lactobacillus plantarum* C2 to plant niches. *Sci. Rep*. **6**, 27392; doi: 10.1038/srep27392 (2016).

## Supplementary Material

Supplementary Information

Supplementary Dataset 1

Supplementary Dataset 2

Supplementary Dataset 3

Supplementary Dataset 4

Supplementary Dataset 5

## Figures and Tables

**Table 1:** Main chemical composition of the culture media.

	CJ	PJ	MRS
pH	5.81 ± 0.02^a^	3.69 ± 0.02^c^	5.71 ± 0.01^b^
Total titratable acidity (mL NaOH 0.1 M 10 ml^−1^)	1.2 ± 0.1^c^	7.4 ± 0.2^a^	4.4 ± 0.1^b^
Soluble solids (Brix)	5.6 ± 0.2^b^	11.1 ± 0.4^a^	5.5 ± 0.1^b^
Total phenols (mM gallic acid eq)	3.0 ± 0.3^b^	5.4 ± 0.2^a^	n.d.
Buffering capacity (mmol HCl pH^−1^ l^−1^)	5.0 ± 0.1^c^	27.0 ± 0.8^a^	20.0 ± 0.6^b^
Free amino acids (mg l^−1^)	1095 ± 37^b^	574 ± 37^c^	3800 ± 61^a^
Glucose (mM)	58 ± 2^c^	206 ± 5^a^	115 ± 4^b^
Fructose (mM)	47 ± 1^b^	212 ± 7^a^	n.d.
Sucrose (mM)	56 ± 2^a^	33 ± 2^b^	n.d.
Malic acid (mM)	29 ± 1^a^	21 ± 1^b^	n.d.
Citric acid (mM)	n.d.	20 ± 1	n.d.

The data are the means of three independent experiments ± standard deviation, analysed in duplicate.

Means within a row with different letters (a–c) are significantly different (*P* < 0.05). n.d., Not detected.

## References

[b1] Ryall B., Eydallin G. & Ferenci T. Culture history and population heterogeneity as determinants of bacterial adaptation: the adaptomics of a single environmental transition. Microbiol Mol Biol Rev 76, 597–625 (2012). 22933562 10.1128/MMBR.05028-11 PMC3429624

[b2] Siezen R. J. . Phenotypic and genomic diversity of *Lactobacillus plantarum* strains isolated from various environmental niches. Environ Microbiol 12, 758–773 (2010). 20002138 10.1111/j.1462-2920.2009.02119.x

[b3] Teusink B. . Analysis of growth of *Lactobacillus plantarum* WCFS1 on a complex medium using a genome-scale metabolic model. J Biol Chem 281, 40041–40048 (2006). 17062565 10.1074/jbc.M606263200

[b4] Kleerebezem M. . Complete genome sequence of *Lactobacillus plantarum* WCFS1. Proc Natl Acad Sci USA 100, 1990–1995 (2003). 12566566 10.1073/pnas.0337704100 PMC149946

[b5] Axelsson L. . Genome sequence of the naturally plasmid-free *Lactobacillus plantarum* strain NC8 (CCUG 61730). J Bacteriol 194, 2391–2392 (2012). 22493200 10.1128/JB.00141-12 PMC3347089

[b6] Siezen R. J. & van Hylckama Vlieg J. E. Genomic diversity and versatility of *Lactobacillus plantarum*, a natural metabolic engineer. Microb Cell Fact 10, S3 (2011). 21995294 10.1186/1475-2859-10-S1-S3 PMC3271238

[b7] Molenaar D. . Exploring *Lactobacillus plantarum* genome diversity by using microarrays. J Bacteriol 187, 6119–6127 (2005). 16109953 10.1128/JB.187.17.6119-6127.2005 PMC1196139

[b8] Bron P. A. . Transcriptomes reveal genetic signatures underlying physiological variations imposed by different fermentation conditions in *Lactobacillus plantarum*. PLos one 7, e38720 (2012). 22802930 10.1371/journal.pone.0038720 PMC3389018

[b9] Leimena M. M. . Comparative analysis of *Lactobacillus plantarum* WCFS1 transcriptomes by using DNA microarray and next-generation sequencing technologies. Appl Environ Microbiol 78, 4141–4148 (2012). 22492454 10.1128/AEM.00470-12 PMC3370542

[b10] Wels M., Overmars L., Francke C., Kleerebezem M. & Siezen R. J. Reconstruction of the regulatory network of *Lactobacillus plantarum* WCFS1 on basis of correlated gene expression and conserved regulatory motifs. Microb Biotechnol 4, 333–344 (2011). 21375715 10.1111/j.1751-7915.2010.00217.x PMC3818992

[b11] Reverón I., Rivas B., Muñoz R. & de Felipe F. L. Genome-wide transcriptomic responses of a human isolate of *Lactobacillus plantarum* exposed to *p*-coumaric acid stress. Mol Nutr Food Res, 56, 1848–1859 (2012). 23065750 10.1002/mnfr.201200384

[b12] de Veen H. . Short- and long-term adaptation to ethanol stress and its cross-protective consequences in *Lactobacillus plantarum*. Appl Environ Microbiol 77, 5247–5256 (2011). 21705551 10.1128/AEM.00515-11 PMC3147428

[b13] Gobbetti M., De Angelis M., Corsetti A. & Di Cagno R. Biochemistry and physiology of sourdough lactic acid bacteria. Trends Food Sci Technol 16, 57–69 (2005).

[b14] Gäenzle M. & Fallador R. Metabolism of oligosaccharides and starch in lactobacilli: a review. Front Microbiol 3, 340 (2012). 23055996 10.3389/fmicb.2012.00340 PMC3458588

[b15] Di Cagno R., Coda R., De Angelis M. & Gobbetti M. Exploitation of vegetables and fruits through lactic acid fermentation. Food Microbiol 33, 1–10 (2013). 23122495 10.1016/j.fm.2012.09.003

[b16] van Baarlen P. . Differential NF-kB pathways induction by *Lactobacillus plantarum* in the duodenum of healthy humans correlating with immune tolerance. Proc Natl Acad Sci USA 106, 2371–2376 (2009). 19190178 10.1073/pnas.0809919106 PMC2650163

[b17] Filannino P. . Metabolic responses of *Lactobacillus plantarum* strains during fermentation and storage of vegetable and fruit juices. Appl Environ Microbiol 80, 2206–2215. (2014). 24487533 10.1128/AEM.03885-13 PMC3993129

[b18] Kahala M., Ahola V., Mäkimattila E., Paulin L. & Joutsjoki V. The use of macroarray as a simple tool to follow the metabolic profile of *Lactobacillus plantarum* during fermentation. Adv Microbiol 4, 996–1016 (2014).

[b19] Siragusa S. . Fermentation and proteome profiles of *Lactobacillus plantarum* strains during growth under food-like conditions. J Proteomics 96, 366–380 (2014). 24231110 10.1016/j.jprot.2013.11.003

[b20] Dallas P. B. . Gene expression levels assessed by oligonucleotide microarray analysis and quantitative real-time RT-PCR–how well do they correlate? BMC genomics 6, 59 (2005). 15854232 10.1186/1471-2164-6-59 PMC1142514

[b21] Sturme M. H. J. . An agr-like two-component regulatory system in *Lactobacillus plantarum* is involved in production of a novel cyclic peptide and regulation of adherence. J Bacteriol 187, 5224–5235 (2005). 16030216 10.1128/JB.187.15.5224-5235.2005 PMC1196011

[b22] Kovács M. . A functional *dlt* operon, encoding proteins required for incorporation of d-alanine in teichoic acids in gram-positive bacteria, confers resistance to cationic antimicrobial peptides in *Streptococcus pneumoniae*. J Bacteriol 188, 5797–5805 (2006). 16885447 10.1128/JB.00336-06 PMC1540085

[b23] Kvint K., Nachin L., Diez A. & Nyström T. The bacterial universal stress protein: function and regulation. Curr Opin Microbiol 6, 140–145 (2003). 12732303 10.1016/s1369-5274(03)00025-0

[b24] Golomb B. L. & Marco M. L. *Lactococcus lactis* metabolism and gene expression during growth on plant tissues. J Bacteriol 197, 371–381 (2015). 25384484 10.1128/JB.02193-14 PMC4272587

[b25] Wang X., Kimet al. Cryptic prophages help bacteria cope with adverse environments. Nat Commun 1, 147 (2010). 21266997 10.1038/ncomms1146 PMC3105296

[b26] Kang T. S., Korber D. R. & Tanaka T. Contributions of citrate in redox potential maintenance and ATP production: metabolic pathways and their regulation in *Lactobacillus panis* PM1. Appl Microbiol Biotechnol 97, 8693–8703 (2013). 23912115 10.1007/s00253-013-5108-2

[b27] Smeianov V. V. . Comparative high-density microarray analysis of gene expression during growth of *Lactobacillus helveticus* in milk versus rich culture medium. Appl Environ Microbiol 73, 2661–2672 (2007). 17322329 10.1128/AEM.00005-07 PMC1855617

[b28] Rodionov D. A. . Transcriptional regulation of NAD metabolism in bacteria: NrtR family of Nudix-related regulators. Nucleic Acids Res 36, 2047–2059 (2008). 18276643 10.1093/nar/gkn047 PMC2330246

[b29] Sturme M. H. J., Francke C., Siezen R. J., de Vos W. M. & Kleerebezem M. Making sense of quorum sensing in lactobacilli: a special focus on *Lactobacillus plantarum* WCFS1. Microbiology 153, 3939–3947 (2007). 18048908 10.1099/mic.0.2007/012831-0

[b30] Grebe T. W. & Stock J. B. The histidine protein kinase superfamily. Adv Microb Physiol 41, 139–227 (1999). 10500846 10.1016/s0065-2911(08)60167-8

[b31] Weinrauch Y., Penchev R., Dubnau E., Smith I. & Dubnau D. A *Bacillus subtilis* regulatory gene product for genetic competence and sporulation resembles sensor protein members of the bacterial two- component signal-transduction systems. Genes Dev 4, 860–872 (1990). 2116363 10.1101/gad.4.5.860

[b32] Siezen R. . *Lactobacillus plantarum* gene clusters encoding putative cell-surface protein complexes for carbohydrate utilization are conserved in specific gram-positive bacteria. BMC Genomics 7, 126 (2006). 16723015 10.1186/1471-2164-7-126 PMC1534035

[b33] Kleerebezem M. . The extracellular biology of the lactobacilli. FEMS Microbiol Rev 34, 199–230 (2010). 20088967 10.1111/j.1574-6976.2010.00208.x

[b34] Zhou M., Theunissen D., Wels M. & Siezen R. J. LAB-Secretome: a genome-scale comparative analysis of the predicted extracellular and surface associated proteins of Lactic Acid Bacteria. BMC Genomics 11, 651 (2010). 21092245 10.1186/1471-2164-11-651 PMC3017865

[b35] Liang W. D. . Gene Expression Profiling of *Clostridium botulinum* under Heat Shock Stress. BioMed Res Int 2013, 760904 (2013). 24195079 10.1155/2013/760904 PMC3806222

[b36] Cui L., Neoh H. M., Iwamoto A. & Hiramatsu K. Coordinated phenotype switching with large-scale chromosome flip-flop inversion observed in bacteria. Proc Natl Acad Sci USA 109, E1647–E1656 (2012). 22645353 10.1073/pnas.1204307109 PMC3382547

[b37] Plumed-Ferrer C. . Comparative study of sugar fermentation and protein expression patterns of two *Lactobacillus plantarum* strains grown in three different media. Appl Environ Microbiol 74, 5349–5358 (2008). 18567686 10.1128/AEM.00324-08 PMC2546631

[b38] Wegkamp A. . Physiological responses to folate overproduction in *Lactobacillus plantarum* WCFS1. Microb Cell Fact 9, 100 (2010). 21167023 10.1186/1475-2859-9-100 PMC3014895

[b39] Phadtare S. Recent developments in bacterial cold-shock response. Curr Issues Mol Biol 6, 125–36 (2004). 15119823

[b40] Rodionov D. A., Vitreschak A. G., Mironov A. A. & Gelfand M. S. Comparative genomics of the methionine metabolism in Gram-positive bacteria: a variety of regulatory systems. Nucleic Acids Res 32, 3340–3353 (2004). 15215334 10.1093/nar/gkh659 PMC443535

[b41] Zhai Z. . Proteomic characterization of the acid tolerance response in *Lactobacillus delbrueckii* subsp. *bulgaricus* CAUH1 and functional identification of a novel acid stress-related transcriptional regulator Ldb0677. Environ Microbiol 16, 1524–1537 (2014). 24131507 10.1111/1462-2920.12280

[b42] van Bokhorst-van de Veen H. . Modulation of *Lactobacillus plantarum* gastrointestinal robustness by fermentation conditions enables identification of bacterial robustness markers. PLos one 7, 39053 (2012). 10.1371/journal.pone.0039053 PMC3389004 22802934

[b43] Zhang Y. M. & Rock C. O. Membrane lipid homeostasis in bacteria. Nat Rev Microiol 6, 222–233 (2008). 10.1038/nrmicro1839 18264115

[b44] Crompton M. J. . Small changes in environmental parameters lead to alterations in antibiotic resistance, cell morphology and membrane fatty acid composition in *Staphylococcus lugdunensis*. PLos one 9, e92296 (2014). 24714666 10.1371/journal.pone.0092296 PMC3979647

[b45] Wall T. . The early response to acid shock in *Lactobacillus reuteri* involves the ClpL chaperone and a putative cell wall-altering esterase. Appl Environ Microbiol 73, 3924–3935 (2007). 17449683 10.1128/AEM.01502-06 PMC1932720

[b46] Yanofsky C. RNA-based regulation of genes of tryptophan synthesis and degradation, in bacteria. RNA 13, 1141–1154 (2007). 17601995 10.1261/rna.620507 PMC1924887

[b47] Koponen J. . Effect of acid stress on protein expression and phosphorylation in *Lactobacillus rhamnosus* GG. J Proteomics 75, 1357–1374 (2012). 22119544 10.1016/j.jprot.2011.11.009

[b48] Cotter P. D. & Hill C. Surviving the acid test: responses of gram-positive bacteria to low pH. Microbiol Mol Biol Rev 67, 429–453 (2003). 12966143 10.1128/MMBR.67.3.429-453.2003 PMC193868

[b49] Budin-Verneuil A., Pichereau V., Auffray Y., Ehrlich D. S. & Maguin E. Proteomic characterization of the acid tolerance response in *Lactococcus lactis* MG1363. Proteomics 5, 4794–4807 (2005). 16237734 10.1002/pmic.200401327

[b50] Caldas T. D., Laalami S. & Richarme G. Chaperone properties of bacterial elongation factor EF-G and initiation factor IF2. J Biol Chem 275, 855–860 (2000). 10625618 10.1074/jbc.275.2.855

[b51] Postma P. W., Lengeler J. W. & Jacobson G. R. Phosphoenolpyruvate: carbohydrate phosphotransferase systems of bacteria. Microbiol Rev 57, 543–594 (1993). 8246840 10.1128/mr.57.3.543-594.1993 PMC372926

[b52] Di Cagno R. . Selection and use of autochthonous mixed starter for lactic acid fermentation of carrots, French beans or marrows. Int J Food Microbiol 127, 220–228 (2008). 18710789 10.1016/j.ijfoodmicro.2008.07.010

[b53] Zwietering M. H., Jongeberger I., Roumbouts F. M. & Van’t Riet K. Modelling of bacterial growth curve. Appl Environ Microbiol 56, 1875–1881 (1990). 16348228 10.1128/aem.56.6.1875-1881.1990 PMC184525

[b54] Bean C. . Ankrd2 is a modulator of NF-κB-mediated inflammatory responses during muscle differentiation. Cell Death Dis, 5, e1002 (2014). 24434510 10.1038/cddis.2013.525 PMC4040671

[b55] Cline M. S. . Integration of biological networks and gene expression data using Cytoscape. Nat Protoc 2, 2366–2382 (2007). 17947979 10.1038/nprot.2007.324 PMC3685583

[b56] Kanehisa M. . Data, information, knowledge and principle: back to metabolism in KEGG. Nucleic Acids Res 42, D199–D205 (2014). 24214961 10.1093/nar/gkt1076 PMC3965122

[b57] Kanehisa M. & Goto S. KEGG: Kyoto Encyclopedia of Genes and Genomes. Nucleic Acids Res 28, 27–30 (2000). 10592173 10.1093/nar/28.1.27 PMC102409

[b58] Klukas C. & Schreiber F. Dynamic exploration and editing of KEGG pathway diagrams. Bioinformatics 23, 344–350 (2007). 17142815 10.1093/bioinformatics/btl611

[b59] Livak K. J. & Schmittgen T. D. Analysis of relative gene expression data using real-time quantitative PCR and the 2^−ΔΔCT^ method. Methods 25, 402–408 (2001). 11846609 10.1006/meth.2001.1262

[b60] Peirson S. N., Butler J. N. & Foster R. G. Experimental validation of novel and conventional approaches to quantitative real-time PCR data analysis. Nucleic Acids Res 31, e73 (2003). 12853650 10.1093/nar/gng073 PMC167648

