# Supplementary material for: Transcriptional reprogramming and phenotypic switching associated with the adaptation of Lactobacillus plantarum C2 to plant niches
Source: Sci Rep. 2016 Jun 7;6:27392. doi: 10.1038/srep27392 (PMC4895336; doi:10.1038/srep27392)
Supplement: Supplementary Information [file srep27392-s1.pdf]

Transcriptional reprogramming and phenotypic switching associated with the adaptation of *Lactobacillus plantarum* C2 to plant niches

Pasquale Filannino[a], Raffaella Di Cagno[a]*, Carmine Crecchio[a], Caterina De Virgilio[b], Maria De Angelis[a], Marco Gobbetti[a]

[a]Department of Plant, Soil and Food Sciences, University of Bari A. Moro, Via Amendola 165/a, 70126 Bari, Italy

[b]Department of Biosciences, Biotechnologies and Biopharmaceutics, University of Bari, Via Orabona, 4, 70126 Bari, Italy

Table of Contents:

- Supplementary Figures S1-S8
- Supplementary Tables S1- S6

Figure S1. Experimental design and flowchart.

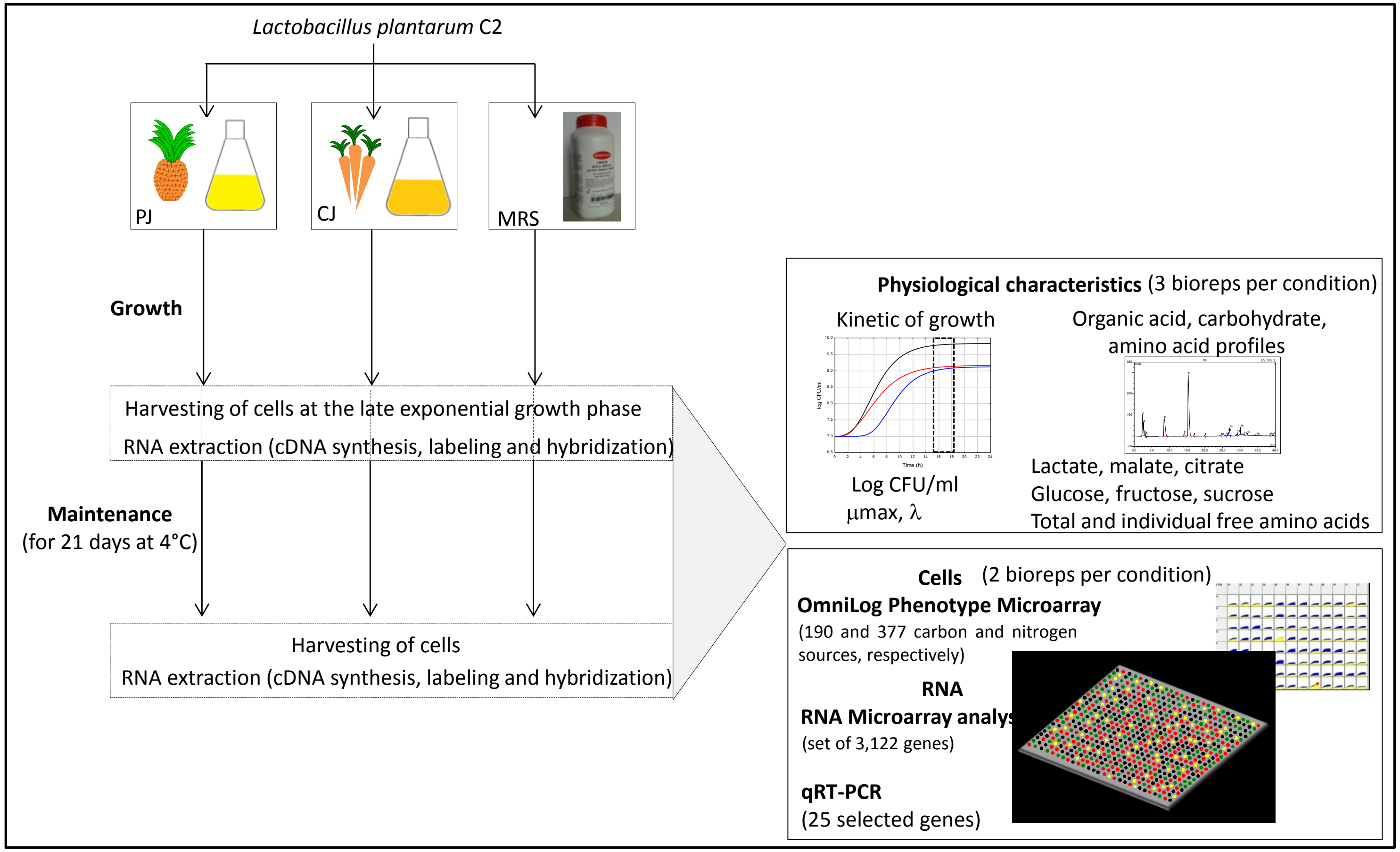

Lactobacillus plantarum C2 was cultivated in carrot and pineapple juice, chosen as model systems representative of vegetables and fruits, respectively. Samples derived from the late exponential growth phase and maintenance were assessed at the molecular level through genome-wide transcriptome analysis, and the phenotypic microarray and physiological characteristics were determined in parallel.

Figure S2.

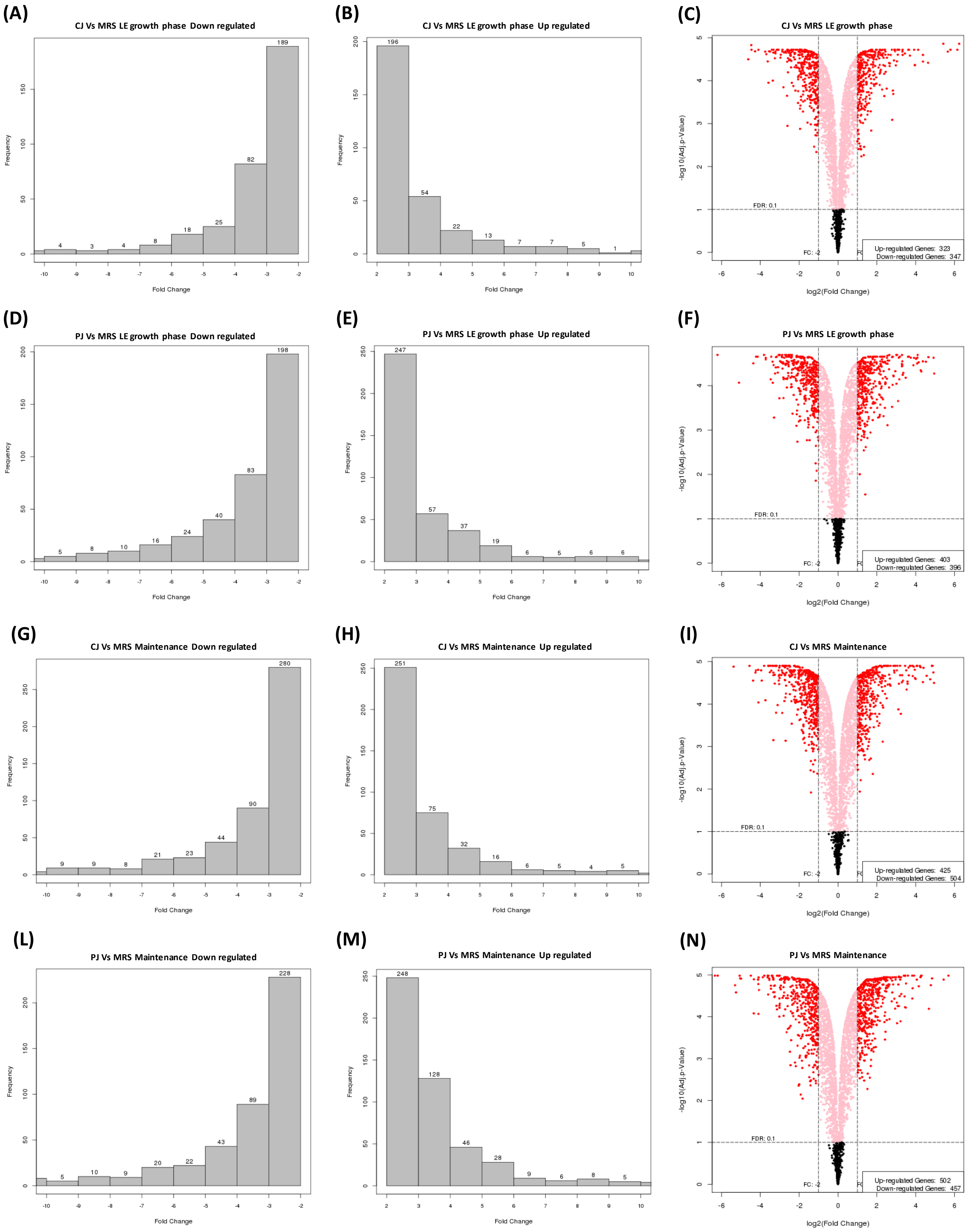
(A)
CJ Vs MRS LE growth phase Down regulated
Frequency
Fold Change
4
3
4
8
18
25
82
189
(B)
CJ Vs MRS LE growth phase Up regulated
Frequency
Fold Change
196
54
22
13
7
7
5
1
(C)
CJ Vs MRS LE growth phase
-log10(Adj.p-Value)
log2(Fold Change)
FDR: 0.1
FC: -2
Up-regulated Genes: 323
Down-regulated Genes: 347
(D)
PJ Vs MRS LE growth phase Down regulated
Frequency
Fold Change
5
8
10
16
24
40
83
198
(E)
PJ Vs MRS LE growth phase Up regulated
Frequency
Fold Change
247
57
37
19
6
5
6
6
(F)
PJ Vs MRS LE growth phase
-log10(Adj.p-Value)
log2(Fold Change)
FDR: 0.1
FC: -2
Up-regulated Genes: 403
Down-regulated Genes: 396
(G)
CJ Vs MRS Maintenance Down regulated
Frequency
Fold Change
9
9
8
21
23
44
90
280
(H)
CJ Vs MRS Maintenance Up regulated
Frequency
Fold Change
251
75
32
16
6
5
4
5
(I)
CJ Vs MRS Maintenance
-log10(Adj.p-Value)
log2(Fold Change)
FDR: 0.1
FC: -2
Up-regulated Genes: 425
Down-regulated Genes: 504
(L)
PJ Vs MRS Maintenance Down regulated
Frequency
Fold Change
5
10
9
20
22
43
89
228
(M)
PJ Vs MRS Maintenance Up regulated
Frequency
Fold Change
248
128
46
28
9
6
8
5
(N)
PJ Vs MRS Maintenance
-log10(Adj.p-Value)
log2(Fold Change)
FDR: 0.1
FC: -2
Up-regulated Genes: 502
Down-regulated Genes: 457

Histograms showing fold-change ratios and frequencies of up-regulated and down-regulated genes during the late exponential (LE) growth phase (16 or 18 h at 30°C) (A, B, D, and E) and the maintenance period (21 days at 4°C) (G, H, L, and M) in carrot (CJ) and pineapple (PJ) juices compared to MRS medium. Volcano plots showing gene expression in *L. plantarum* C2 during the LE growth phase (C and F) and the maintenance period (I and N) in CJ and PJ compared to MRS medium. The x-axis represents the differential gene expression profiles plotted as fold-induction ratios on a log-2 scale. The y-axis indicates the statistical significance of the differences in expression (p-value as calculated by ANOVA) on a $\log_{10}$ scale.

Figure S3.

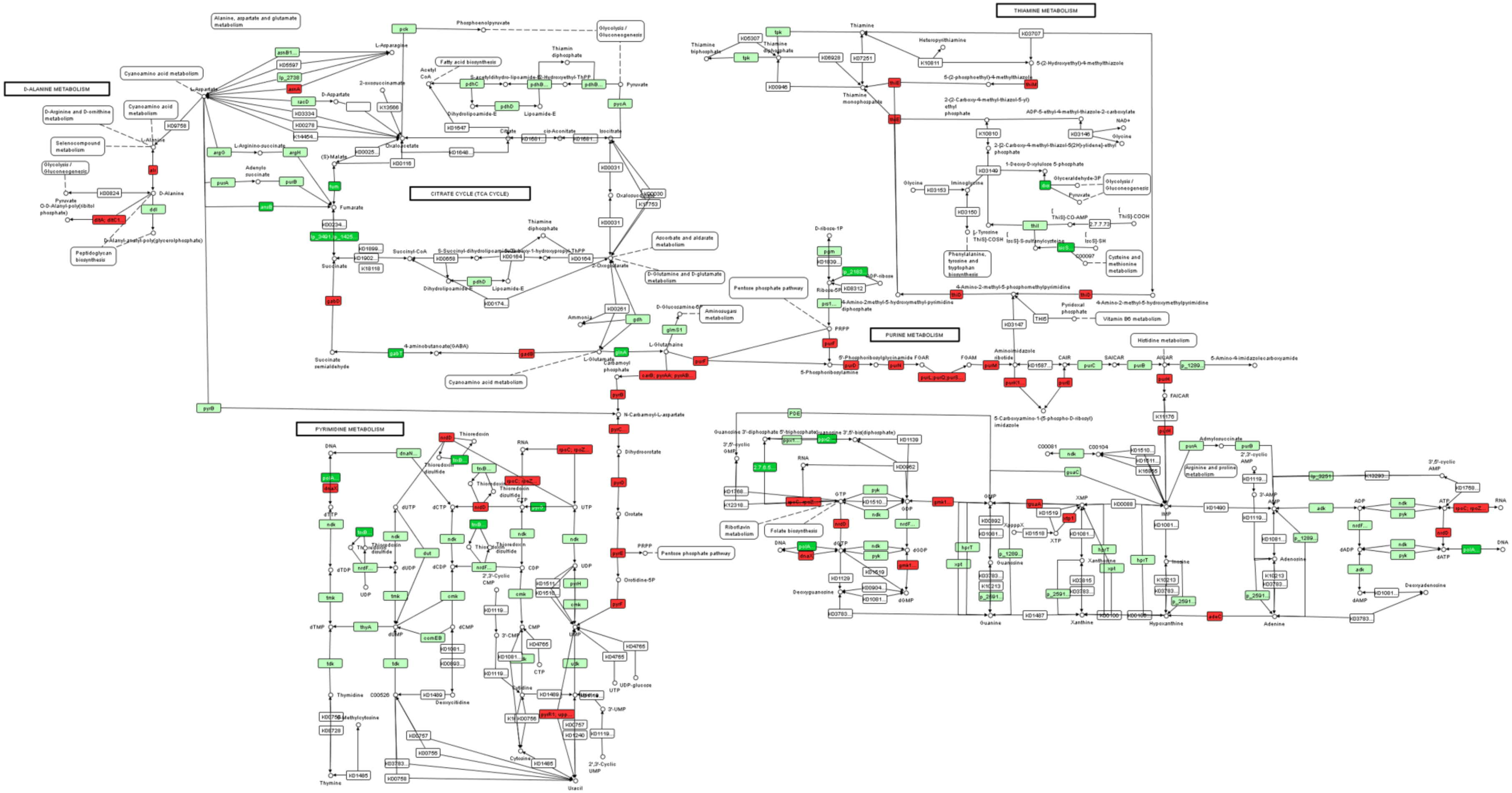
D-ALANINE METABOLISM
CITRATE CYCLE (TCA CYCLE)
THIAMINE METABOLISM
PURINE METABOLISM
PYRIMIDINE METABOLISM

Integrated pathways of *Lactobacillus plantarum* C2 during the late exponential growth phase (16 h at 30°C) in carrot juice (CJ) compared to MRS medium. A screenshot of the DAVID pathway analysis of the set of differentially expressed genes is shown. The nodes for genes encoding enzymes are coloured lime green if up-regulated and red if down-regulated. Other enzyme nodes appear in pale green. Substrate nodes appear in white. Multiple up/down-regulated genes are separated by semicolons. Analytical modules are provided in Dataset S4.

Figure S4.

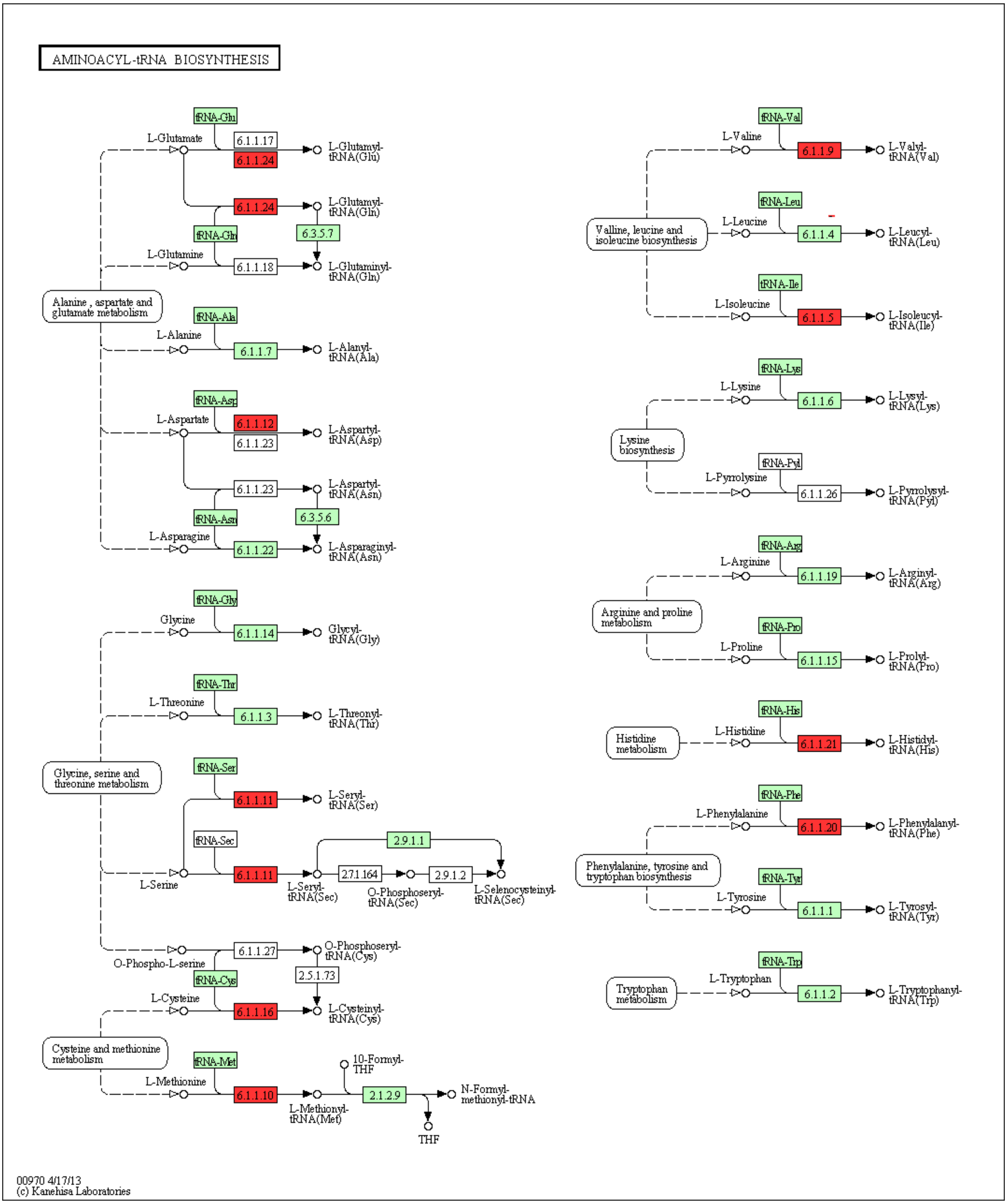
AMINOACYL-tRNA BIOSYNTHESIS
tRNA-Glu
L-Glutamate
6.1.1.17
6.1.1.24
L-Glutamyl-tRNA(Glu)
6.1.1.24
L-Glutamyl-tRNA(Gln)
6.3.5.7
tRNA-Gln
L-Glutamine
6.1.1.18
L-Glutaminyl-tRNA(Gln)
Alanine, aspartate and glutamate metabolism
tRNA-Ala
L-Alanine
6.1.1.7
L-Alanyl-tRNA(Ala)
tRNA-Asp
L-Aspartate
6.1.1.12
6.1.1.23
L-Aspartyl-tRNA(Asp)
6.1.1.23
L-Aspartyl-tRNA(Asn)
6.3.5.6
tRNA-Asn
L-Asparagine
6.1.1.22
L-Asparaginyl-tRNA(Asn)
tRNA-Gly
Glycine
6.1.1.14
Glycyl-tRNA(Gly)
tRNA-Thr
L-Threonine
6.1.1.3
L-Threonyl-tRNA(Thr)
Glycine, serine and threonine metabolism
tRNA-Ser
6.1.1.11
L-Seryl-tRNA(Ser)
tRNA-Sec
2.9.1.1
6.1.1.11
2.7.1.164
2.9.1.2
L-Serine
L-Seryl-tRNA(Sec)
O-Phosphoseryl-tRNA(Sec)
L-Selenocysteinyl-tRNA(Sec)
6.1.1.27
O-Phosphoseryl-tRNA(Cys)
O-Phospho-L-serine
2.5.1.73
tRNA-Cys
L-Cysteine
6.1.1.16
L-Cysteinyl-tRNA(Cys)
Cysteine and methionine metabolism
tRNA-Met
L-Methionine
6.1.1.10
10-Formyl-THF
2.1.2.9
N-Formyl-methionyl-tRNA
L-Methionyl-tRNA(Met)
THF
tRNA-Val
L-Valine
6.1.1.9
L-Valyl-tRNA(Val)
tRNA-Leu
Valline, leucine and isoleucine biosynthesis
L-Leucine
6.1.1.4
L-Leucyl-tRNA(Leu)
tRNA-Ile
L-Isoleucine
6.1.1.5
L-Isoleucyl-tRNA(Ile)
tRNA-Lys
L-Lysine
6.1.1.6
L-Lysyl-tRNA(Lys)
Lysine biosynthesis
tRNA-Pyl
L-Pyrrolysine
6.1.1.26
L-Pyrrolysyl-tRNA(Pyl)
tRNA-Arg
L-Arginine
6.1.1.19
L-Arginyl-tRNA(Arg)
Arginine and proline metabolism
tRNA-Pro
L-Proline
6.1.1.15
L-Prolyl-tRNA(Pro)
tRNA-His
Histidine metabolism
L-Histidine
6.1.1.21
L-Histidyl-tRNA(His)
tRNA-Phe
L-Phenylalanine
6.1.1.20
L-Phenylalanyl-tRNA(Phe)
Phenylalanine, tyrosine and tryptophan biosynthesis
tRNA-Tyr
L-Tyrosine
6.1.1.1
L-Tyrosyl-tRNA(Tyr)
tRNA-Trp
Tryptophan metabolism
L-Tryptophan
6.1.1.2
L-Tryptophanyl-tRNA(Trp)
00970 4/17/13
(c) Kanehisa Laboratories

The aminoacyl-tRNA biosynthesis pathway in *Lactobacillus plantarum* C2 during the late exponential growth phase in carrot juice (CJ) compared to MRS medium. A screenshot of the DAVID pathway analysis of the set of differentially expressed genes is shown. The nodes for genes encoding enzymes are coloured lime green if up-regulated and red if down-regulated. Other enzyme nodes appear in pale green. Substrate nodes appear in white. Analytical modules are provided in Dataset S4.

Figure S5.

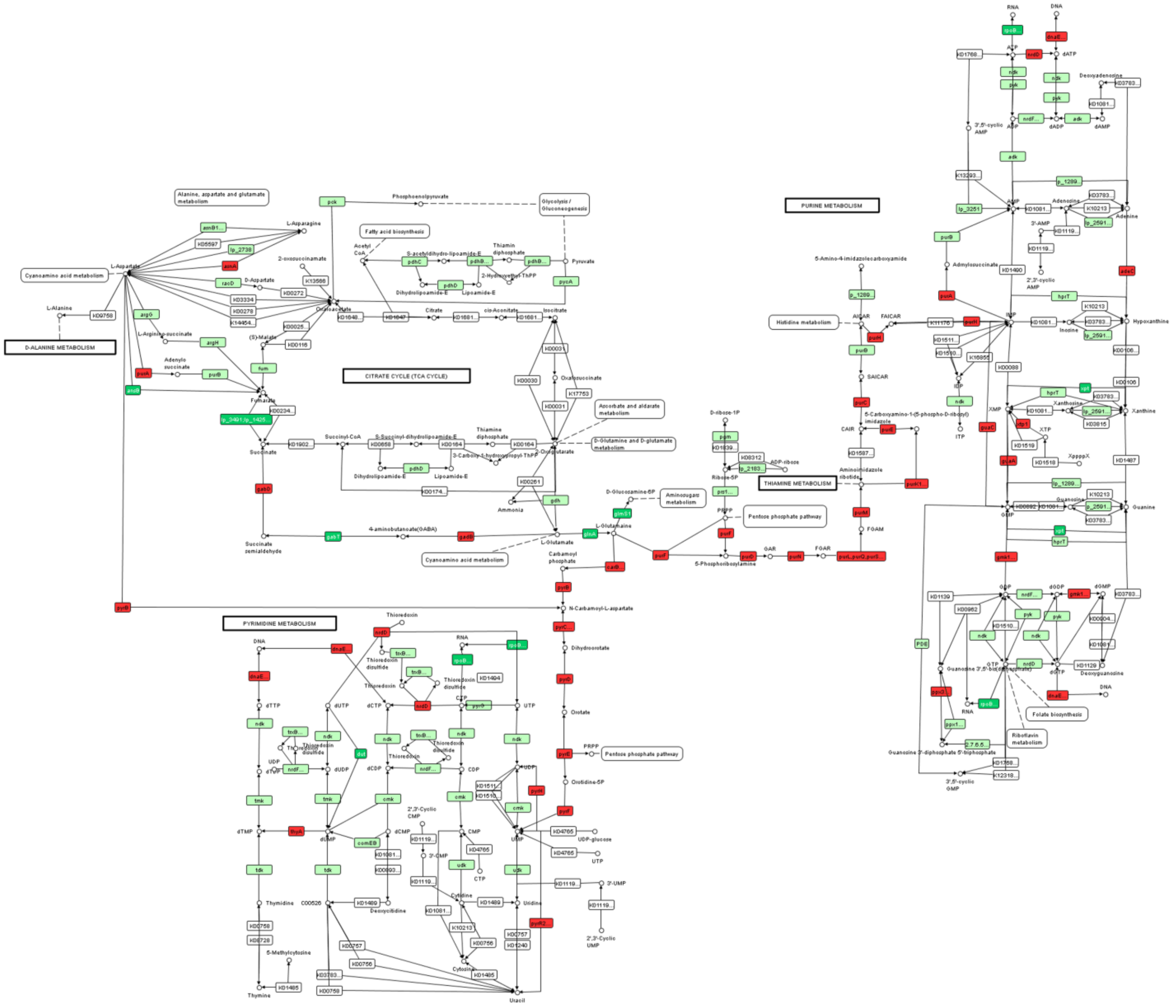
PURINE METABOLISM
CITRATE CYCLE (TCA CYCLE)
D-ALANINE METABOLISM
THIAMINE METABOLISM
PYRIMIDINE METABOLISM

Integrated pathways of *Lactobacillus plantarum* C2 during maintenance (21 days at 4°C) in carrot juice (CJ) compared to MRS medium. A screenshot of the DAVID pathway analysis of the set of differentially expressed genes is shown. The nodes for genes encoding enzymes are coloured lime green if up-regulated and red if down-regulated. Other enzyme nodes appear in pale green. Substrate nodes appear in white. Multiple up/down-regulated genes are separated by semicolons. Analytical modules are provided in Dataset S4.

Figure S6.

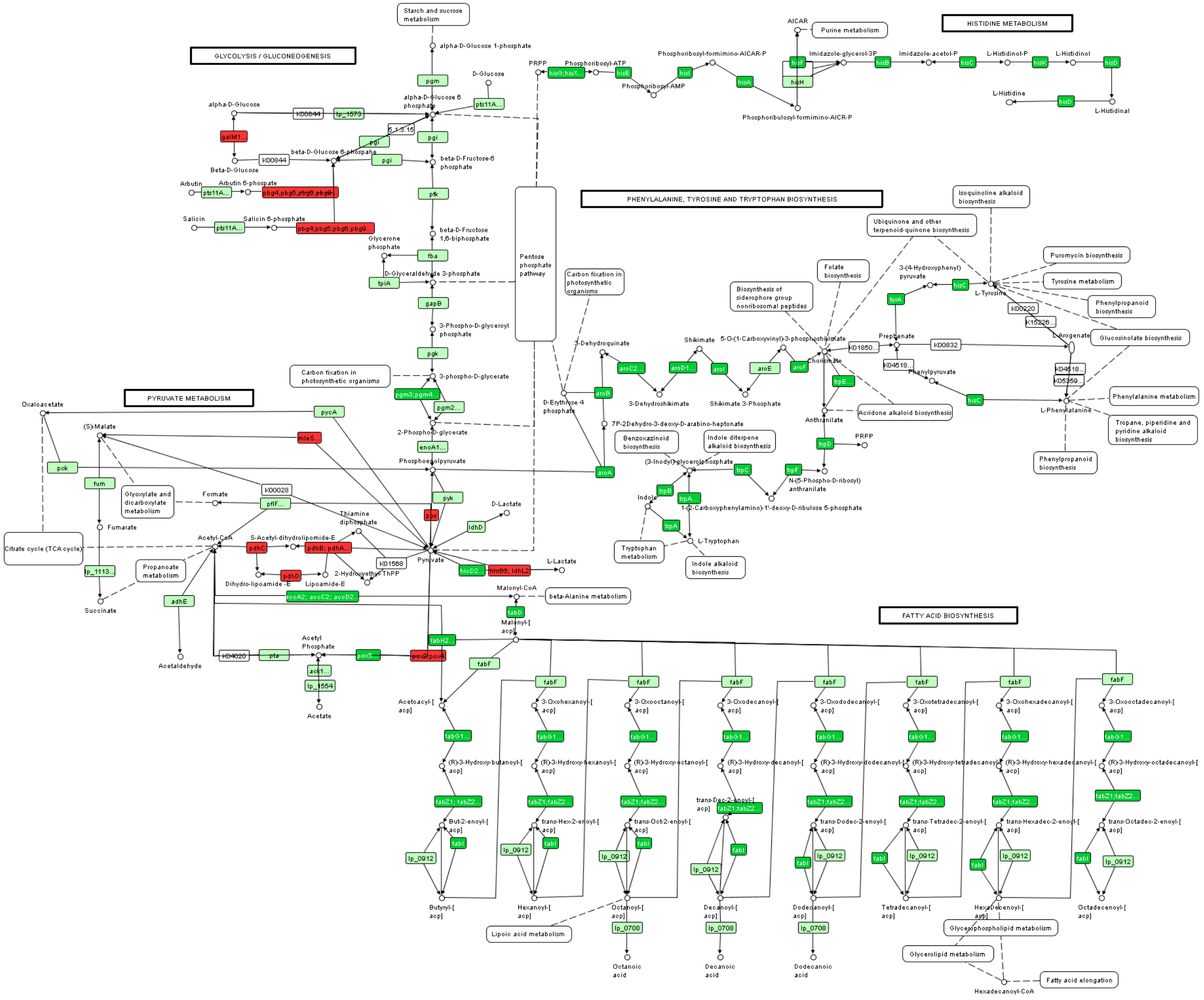

GLYCOLYSIS / GLUCONEOGENESIS
Starch and sucrose metabolism
alpha-D-Glucose 1-phosphate
pgm
D-Glucose
alpha-D-Glucose 6 phosphate
alpha-D-Glucose
beta-D-Glucose 6-phosphate
Beta-D-Glucose
Arbutin
Arbutin 6-phosphate
Salicin
Salicin 6-phosphate
pgi
pfk
beta-D-Fructose-6 phosphate
beta-D-Fructose 1,6-biphosphate
Glycerone phosphate
fba
tpiA
D-Glyceraldehyde 3-phosphate
gapB
3-Phospho-D-glyceroyl phosphate
pgk
3-phospho-D-glycerate
2-Phospho-D-glycerate
enoA1...
Phosphoenolpyruvate
pyk
Pentose phosphate pathway
Carbon fixation in photosynthetic organisms
PYRUVATE METABOLISM
Oxaloacetate
(S)-Malate
Fumarate
Succinate
Glyoxylate and dicarboxylate metabolism
Citrate cycle (TCA cycle)
Propanoate metabolism
Formate
Acetyl-CoA
S-Acetyl-dihydrolipoamide-E
Thiamine diphosphate
2-Hydroxyethyl-ThPP
Dihydro-lipoamide-E
Lipoamide-E
Pyruvate
D-Lactate
L-Lactate
Malonyl-CoA
beta-Alanine metabolism
Acetaldehyde
Acetyl Phosphate
Acetate
PHENYLALANINE, TYROSINE AND TRYPTOPHAN BIOSYNTHESIS
3-Dehydroquinate
3-Dehydroshikimate
Shikimate
Shikimate 3-Phosphate
5-O-(1-Carboxyvinyl)-3-phosphoshikimate
Chorismate
Prephenate
Phenylpyruvate
L-Arogenate
L-Tyrosine
L-Phenylalanine
Anthranilate
PRPP
N-(5-Phospho-D-ribosyl) anthranilate
1-(2-Carboxyphenylamino)-1'-deoxy-D-ribulose 5-phosphate
(3-Indoyl)-glycerolphosphate
Indole
L-Tryptophan
D-Erythrose 4 phosphate
7P-2Dehydro-3-deoxy-D-arabino-heptonate
Tryptophan metabolism
Indole alkaloid biosynthesis
Benzoxazinoid biosynthesis
Indole diterpene alkaloid biosynthesis
Acridone alkaloid biosynthesis
Folate biosynthesis
Biosynthesis of siderophore group nonribosomal peptides
Ubiquinone and other terpenoid-quinone biosynthesis
Isoquinoline alkaloid biosynthesis
Puromycin biosynthesis
Tyrosine metabolism
Phenylpropanoid biosynthesis
Glucosinolate biosynthesis
Phenylalanine metabolism
Tropane, piperidine and pyridine alkaloid biosynthesis
HISTIDINE METABOLISM
AICAR
Purine metabolism
Phosphoribosyl-ATP
Phosphoribosyl-AMP
Phosphoribosyl-formimino-AICAR-P
Phosphoribulosyl-formimino-AICR-P
Imidazole-glycerol-3P
Imidazole-acetol-P
L-Histidinol-P
L-Histidinol
L-Histidinal
L-Histidine
FATTY ACID BIOSYNTHESIS
Malonyl-[acp]
Acetoacyl-[acp]
(R)-3-Hydroxy-butanoyl-[acp]
But-2-enoyl-[acp]
Butyryl-[acp]
Hexanoyl-[acp]
Octanoyl-[acp]
Decanoyl-[acp]
Dodecanoyl-[acp]
Tetradecanoyl-[acp]
Hexadecanoyl-[acp]
Octadecanoyl-[acp]
Octanoic acid
Decanoic acid
Dodecanoic acid
Lipoic acid metabolism
Glycerophospholipid metabolism
Glycerolipid metabolism
Fatty acid elongation
Hexadecanoyl-CoA
fabF
fabG1...
fabZ1;fabZ2...
fabI
lp_0912
lp_0708

Integrated pathways of *Lactobacillus plantarum* C2 during the late exponential growth phase (18 h at 30°C) in pineapple juice (PJ) compared to MRS medium. A screenshot of the DAVID pathway analysis of the set of differentially expressed genes is shown. The nodes for genes encoding enzymes are coloured lime green if up-regulated and red if down-regulated. Other enzyme nodes appear in pale green. Substrate nodes appear in white. Multiple up- or down-regulated genes are separated by semicolons. Analytical modules are provided in Dataset S4.

Figure S7.

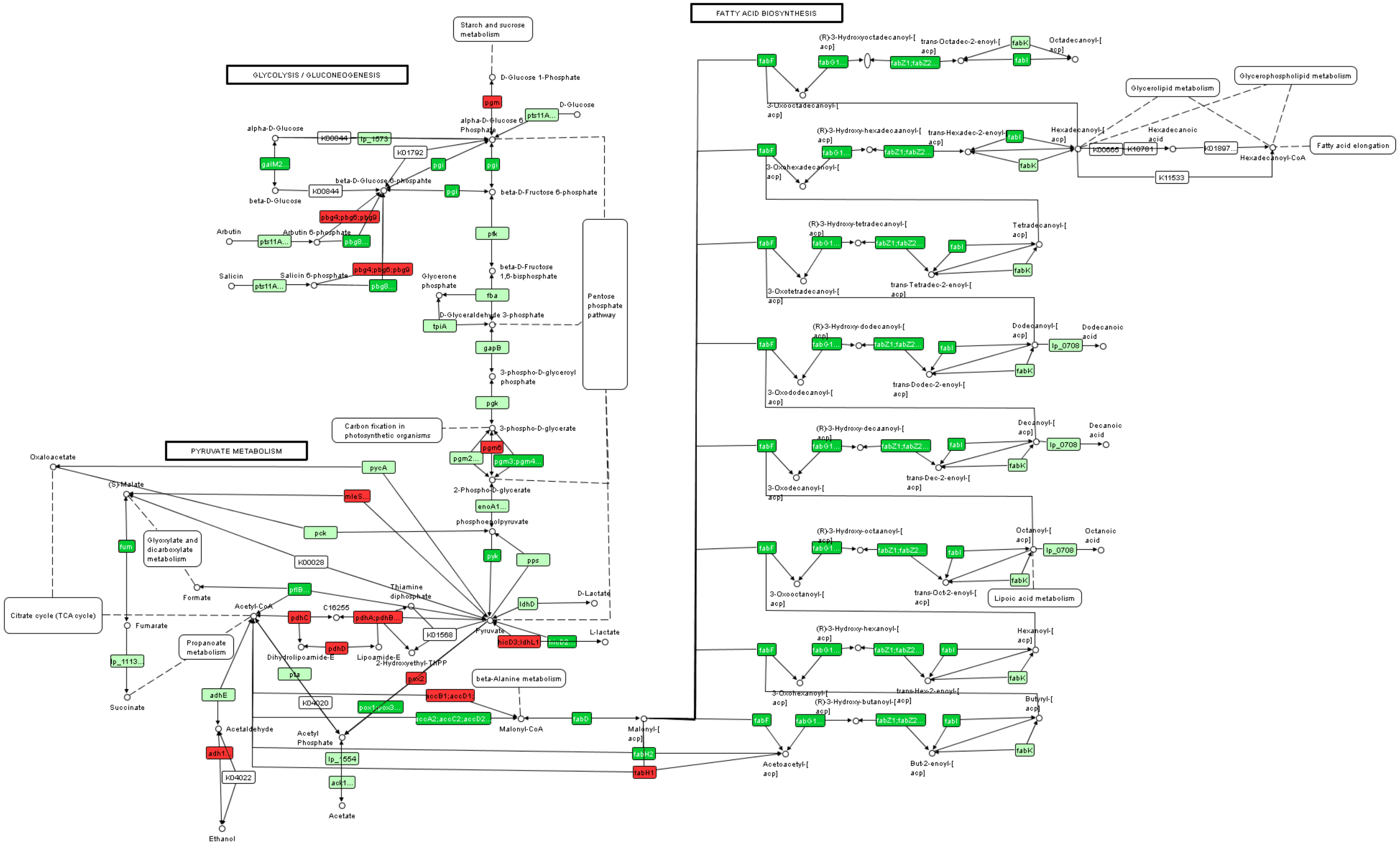

GLYCOLYSIS / GLUCONEOGENESIS
Starch and sucrose metabolism
D-Glucose 1-Phosphate
pgm
D-Glucose
pts11A...
alpha-D-Glucose 6-Phosphate
alpha-D-Glucose
K00844
lp_1573
K01792
galM2...
pgi
beta-D-Glucose 6-phospahte
beta-D-Glucose
beta-D-Fructose 6-phosphate
pbg4;pbg6;pbg9
Arbutin
Arbutin 6-phosphate
pbg8...
Salicin
Salicin 6-phosphate
pfk
beta-D-Fructose 1,6-bisphosphate
Glycerone phosphate
fba
tpiA
D-Glyceraldehyde 3-phosphate
Pentose phosphate pathway
gapB
3-phospho-D-glyceroyl phosphate
pgk
Carbon fixation in photosynthetic organisms
3-phospho-D-glycerate
pgm6
pgm2...
pgm3;pgm4...
2-Phospho-D-glycerate
enoA1...
phosphoenolpyruvate
PYRUVATE METABOLISM
Oxaloacetate
pycA
(S)-Malate
mleS...
pck
fum
Glyoxylate and dicarboxylate metabolism
K00028
pyk
pps
Formate
pflB...
Thiamine diphosphate
ldhD
D-Lactate
Citrate cycle (TCA cycle)
Acetyl-CoA
C16255
pdhC
pdhA;pdhB...
Pyruvate
Fumarate
K01568
hicD3;ldhL1
L-lactate
Propanoate metabolism
pdhD
Dihydrolipoamide-E
Lipoamide-E
2-Hydroxyethyl-ThPP
lp_1113...
pta
poxB
beta-Alanine metabolism
adhE
K04020
accB1;accD1
Succinate
pox1;pox3...
accA2;accC2;accD2...
fabD
Malonyl-CoA
Acetaldehyde
Acetyl Phosphate
Malonyl-[acp]
adh1...
lp_1554
fabH2
K04022
ack1...
fabH1
Acetate
Ethanol
FATTY ACID BIOSYNTHESIS
(R)-3-Hydroxyoctadecanoyl-[acp]
trans-Octadec-2-enoyl-[acp]
Octadecanoyl-[acp]
fabF
fabG1...
fabZ1;fabZ2...
fabK
fabI
3-Oxooctadecanoyl-[acp]
Glycerolipid metabolism
Glycerophospholipid metabolism
(R)-3-Hydroxy-hexadecanoyl-[acp]
trans-Hexadec-2-enoyl-[acp]
Hexadecanoyl-[acp]
Hexadecanoic acid
K00665
K10781
K01897...
Fatty acid elongation
Hexadecanoyl-CoA
3-Oxohexadecanoyl-[acp]
K11533
(R)-3-Hydroxy-tetradecanoyl-[acp]
Tetradecanoyl-[acp]
3-Oxotetradecanoyl-[acp]
trans-Tetradec-2-enoyl-[acp]
(R)-3-Hydroxy-dodecanoyl-[acp]
Dodecanoyl-[acp]
Dodecanoic acid
lp_0708
3-Oxododecanoyl-[acp]
trans-Dodec-2-enoyl-[acp]
(R)-3-Hydroxy-decanoyl-[acp]
Decanoyl-[acp]
Decanoic acid
3-Oxodecanoyl-[acp]
trans-Dec-2-enoyl-[acp]
(R)-3-Hydroxy-octanoyl-[acp]
Octanoyl-[acp]
Octanoic acid
3-Oxooctanoyl-[acp]
trans-Oct-2-enoyl-[acp]
Lipoic acid metabolism
(R)-3-Hydroxy-hexanoyl-[acp]
Hexanoyl-[acp]
3-Oxohexanoyl-[acp]
trans-Hex-2-enoyl-[acp]
(R)-3-Hydroxy-butanoyl-[acp]
Butyryl-[acp]
Acetoacetyl-[acp]
But-2-enoyl-[acp]

Integrated pathways of *Lactobacillus plantarum* C2 during the maintenance period (21 days at 4°C) in pineapple juice (PJ) compared to MRS medium. A screenshot of DAVID pathway analysis of the set of differentially expressed genes is shown. The nodes for genes encoding enzymes are coloured lime green if up-regulated and red if down-regulated. Other enzyme nodes appear in pale green. Substrate nodes appear in white. Multiple up- or down-regulated genes are separated by semicolons. Analytical modules are provided in Dataset S4.

Figure S8.

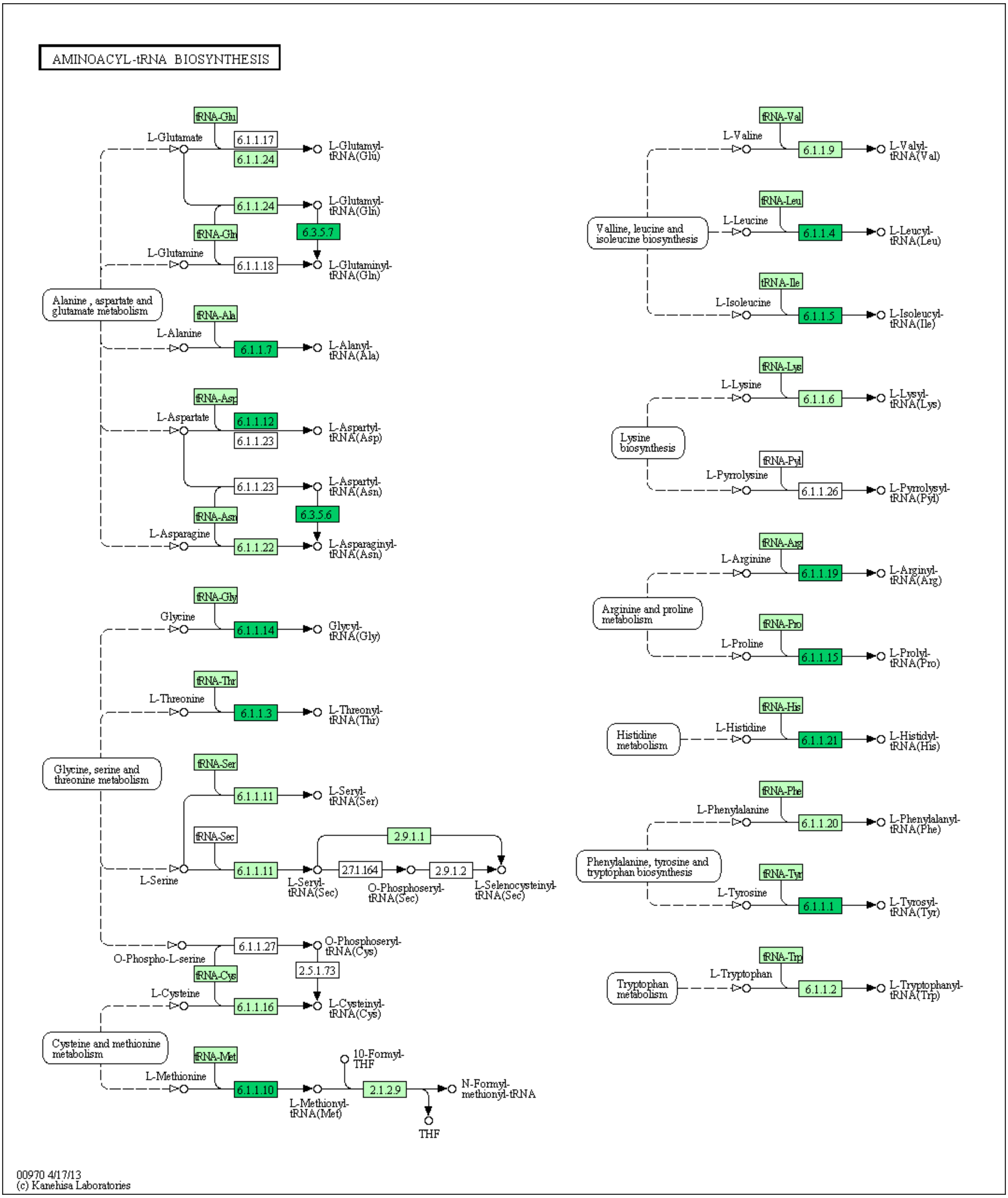

The aminoacyl-tRNA biosynthesis pathway in *Lactobacillus plantarum* C2 during the maintenance period (21 days at 4°C) in pineapple juice (PJ) compared to MRS medium. A screenshot of DAVID

pathway analysis of the set of differentially expressed genes is shown. The nodes for genes encoding enzymes are coloured lime green if up-regulated and red if down-regulated. Other enzyme nodes appear in pale green. Substrate nodes appear in white. Analytical modules are provided in Dataset S4.

Table S1. Phage- and prophage-related genes differentially expressed during the late exponential (LE) growth phase (16 or 18 h at 30°C) and the maintenance period (21 days at 4°C) in carrot (CJ) or pineapple (PJ) juices compared to MRS medium.

	Carrot juice (CJ)			
	**Gene**	**Fold Change**	**Description**	**Accession**
**LE growth phase**	lp_0915	4.82	phage protein	NC_004567.2
	lp_2397	2.08	prophage P2a protein 59; extracellular polysaccharide deacetylase, lipid-anchored	NC_004567.2
	lp_2417	2.55	prophage P2a protein 40; major capsid protein	NC_004567.2
	lp_0681	-11.94	prophage P1 protein 58, lysin	NC_004567.2
	lp_2437	-2.74	prophage P2a protein 20, replication protein DnaD domain	NC_004567.2
	lp_0661	-3.29	prophage P1 protein 38, terminase large subunit TerL	NC_004567.2
	lp_2436	-3.35	prophage P2a protein 21	NC_004567.2
	lp_0644	-3.46	prophage P1 protein 21	NC_004567.2
	lp_2466	-2.09	prophage P2b protein 15, terminase large subunit	NC_004567.2
	lp_2426	-2.67	prophage P2a protein 31; phage ArpU family transcriptional regulator	NC_004567.2
	lp_0682	-21.87	prophage P1 protein 59	NC_004567.2
	lp_2445	-2.73	prophage P2a protein 12	NC_004567.2
	lp_2457	-2.32	prophage P2b protein 24	NC_004567.2
	lp_0656	-3.29	prophage P1 protein 33, phage ArpU family transcriptional regulator	NC_004567.2
	lp_2400	-2.19	prophage P2a protein 57	NC_004567.2
	lp_2399	-5.57	prophage P2a protein 58, holin	NC_004567.2
	lp_0663	-2.06	prophage P1 protein 40, minor head protein	NC_004567.2
	lp_2446	-3.24	prophage P2a protein 11	NC_004567.2
**Maintenance**	lp_2397	12.55	prophage P2a protein 59; extracellular polysaccharide deacetylase, lipid-anchored	NC_004567.2
	lp_2415	2.04	prophage P2a protein 42	NC_004567.2
	lp_2417	3.23	prophage P2a protein 40; major capsid protein	NC_004567.2
	lp_2428	2.85	prophage P2a protein 29	NC_004567.2
	lp_2432	2.40	prophage P2a protein 25	NC_004567.2
	lp_2433	3.21	prophage P2a protein 24; endodeoxyribonuclease RusA-like	NC_004567.2
	lp_2437	3.55	prophage P2a protein 20, replication protein DnaD domain	NC_004567.2

Table S1. Continued

	Gene	Fold Change	Description	Accession
			**Carrot juice (CJ)**	
	**Gene**	**Fold Change**	**Description**	**Accession**
**Maintenance**	lp_2442	3.17	prophage P2a protein 15	NC_004567.2
	lp_0632	2.76	prophage P1 protein 9, phage Cro/CI family transcriptional regulator	NC_004567.2
	lp_0915	3.54	phage protein	NC_004567.2
	lp_2445	2.38	prophage P2a protein 12	NC_004567.2
	lp_2446	2.42	prophage P2a protein 11	NC_004567.2
	lp_2448	2.74	prophage P2a protein 9, phage Cro/CI family transcriptional regulator	NC_004567.2
	lp_2460	2.12	prophage P2b protein 21	NC_004567.2
	lp_2461	3.90	prophage P2b protein 20	NC_004567.2
	lp_2467	2.04	prophage P2b protein 14, terminase small subunit	NC_004567.2
	lp_2474	2.03	prophage P2b protein 7, DNA replication	NC_004567.2
	lp_3381	2.64	prophage P3 protein 9	NC_004567.2
	lp_0661	-5.69	prophage P1 protein 38, terminase large subunit TerL	NC_004567.2
	lp_0663	-3.90	prophage P1 protein 40, minor head protein	NC_004567.2
	lp_0675	-2.38	prophage P1 protein 52, endolysin	NC_004567.2
	lp_0676	-2.43	prophage P1 protein 53, GDSL-like lipase/acylhydrolase family	NC_004567.2
	lp_0682	-2.25	prophage P1 protein 59	NC_004567.2
	lp_2399	-2.04	prophage P2a protein 58, holin	NC_004567.2
	lp_2457	-2.51	prophage P2b protein 24	NC_004567.2
			**Pineapple juice (PJ)**	
	**Gene**	**Fold Change**	**Description**	**Accession**
**LE growth phase**	lp_0643	-2.37	prophage P1 protein 20	NC_004567.2
	lp_0644	-10.32	prophage P1 protein 21	NC_004567.2
	lp_0646	-2.22	prophage P1 protein 23	NC_004567.2
	lp_0656	-6.97	prophage P1 protein 33, phage ArpU family transcriptional regulator	NC_004567.2
	lp_0661	-5.78	prophage P1 protein 38, terminase large subunit TerL	NC_004567.2
	lp_0662	-2.75	prophage P1 protein 39, portal protein	NC_004567.2
	lp_0663	-2.68	prophage P1 protein 40, minor head protein	NC_004567.2

Table S1. Continued

	Pineapple juice (PJ)			
	**Gene**	**Fold Change**	**Description**	**Accession**
**LE growth phase**	lp_0681	-2.88	prophage P1 protein 58, lysin	NC_004567.2
	lp_0682	-2.22	prophage P1 protein 59	NC_004567.2
	lp_2399	-2.39	prophage P2a protein 58, holin	NC_004567.2
	lp_2426	-6.41	prophage P2a protein 31; phage ArpU family transcriptional regulator	NC_004567.2
	lp_2433	-4.26	prophage P2a protein 24; endodeoxyribonuclease RusA-like	NC_004567.2
	lp_2434	-2.35	prophage P2a protein 23	NC_004567.2
	lp_2436	-9.93	prophage P2a protein 21	NC_004567.2
	lp_2437	-6.81	prophage P2a protein 20, replication protein DnaD domain	NC_004567.2
	lp_2442	-8.05	prophage P2a protein 15	NC_004567.2
	lp_2445	-5.92	prophage P2a protein 12	NC_004567.2
	lp_2446	-7.32	prophage P2a protein 11	NC_004567.2
	lp_2455	-2.36	prophage P2a protein 2, integrase	NC_004567.2
	lp_2457	-2.37	prophage P2b protein 24	NC_004567.2
	lp_2466	-2.38	prophage P2b protein 15, terminase large subunit	NC_004567.2
	lp_2472	-2.53	prophage P2b protein 9	NC_004567.2
	lp_2473	-2.36	prophage P2b protein 8, helicase	NC_004567.2
	lp_2480	-2.34	prophage P2b protein 1, integrase	NC_004567.2
**Maintenance**	lp_2397	2.37	prophage P2a protein 59; extracellular polysaccharide deacetylase, lipid-anchored	NC_004567.2
	lp_0915	2.80	phage protein	NC_004567.2
	lp_0643	-2.93	prophage P1 protein 20	NC_004567.2
	lp_0644	-9.51	prophage P1 protein 21	NC_004567.2
	lp_0646	-2.76	prophage P1 protein 23	NC_004567.2
	lp_0656	-10.61	prophage P1 protein 33, phage ArpU family transcriptional regulator	NC_004567.2
	lp_0661	-3.76	prophage P1 protein 38, terminase large subunit TerL	NC_004567.2
	lp_0662	-2.31	prophage P1 protein 39, portal protein	NC_004567.2
	lp_0663	-2.51	prophage P1 protein 40, minor head protein	NC_004567.2
	lp_0681	-3.43	prophage P1 protein 58, lysin	NC_004567.2

Table S1. Continued

	Pineapple juice (PJ)			
	**Gene**	**Fold Change**	**Description**	**Accession**
**Maintenance**	lp_0682	-10.37	prophage P1 protein 59	NC_004567.2
	lp_2399	-2.67	prophage P2a protein 58, holin	NC_004567.2
	lp_2417	-2.62	prophage P2a protein 40; major capsid protein	NC_004567.2
	lp_2426	-8.80	prophage P2a protein 31; phage ArpU family transcriptional regulator	NC_004567.2
	lp_2434	-2.91	prophage P2a protein 23	NC_004567.2
	lp_2436	-10.91	prophage P2a protein 21	NC_004567.2
	lp_2437	-6.83	prophage P2a protein 20, replication protein DnaD domain	NC_004567.2
	lp_2442	-12.30	prophage P2a protein 15	NC_004567.2
	lp_2445	-14.63	prophage P2a protein 12	NC_004567.2
	lp_2446	-13.86	prophage P2a protein 11	NC_004567.2
	lp_2457	-5.12	prophage P2b protein 24	NC_004567.2

Table S2. Cell density (log CFU/ml) and pH values during the growth (24 h at 30°C) and the maintenance period (21 days at 4°C) in carrot juice (CJ), pineapple juice (PJ), and MRS broth.

		**Time (h) at 30°C**													**Time (days) at 4°C**			
		**0**	**2**	**4**	**6**	**8**	**10**	**12**	**14**	**16**	**18**	**20**	**22**	**24**	**5**	**10**	**15**	**21**
**CJ**	log CFU/ml	7.04 ± 0.03	6.98 ± 0.04	7.44 ± 0.02	8.10 ± 0.05	8.45 ± 0.04	8.76 ± 0.02	8.97 ± 0.03	9.00 ± 0.06	9.10 ± 0.05	9.15 ± 0.03	9.16 ± 0.04	9.21 ± 0.06	9.20 ± 0.05	9.12 ± 0.04	9.02 ± 0.02	8.85 ± 0.05	8.68 ± 0.03
	pH	5.81 ± 0.02	5.75 ± 0.02	5.70 ± 0.02	5.61 ± 0.01	5.51 ± 0.02	5.21 ± 0.02	4.91 ± 0.03	4.50 ± 0.01	4.26 ± 0.01	4.24 ± 0.02	4.25 ± 0.03	4.23 ± 0.03	4.19 ± 0.02	4.10 ± 0.02	4.08 ± 0.01	4.06 ± 0.03	4.02 ± 0.02
**PJ**	log CFU/ml	7.02 ± 0.03	6.98 ± 0.04	7.01 ± 0.02	7.16 ± 0.04	7.71 ± 0.04	8.28 ± 0.05	8.68 ± 0.04	8.91 ± 0.03	9.05 ± 0.03	9.08 ± 0.05	9.09 ± 0.06	9.11 ± 0.04	9.13 ± 0.06	8.83 ± 0.02	8.64 ± 0.04	8.51 ± 0.04	8.35 ± 0.03
	pH	3.69 ± 0.02	3.67 ± 0.01	3.66 ± 0.02	3.65 ± 0.01	3.58 ± 0.02	3.56 ± 0.03	3.47 ± 0.04	3.43 ± 0.02	3.40 ± 0.02	3.35 ± 0.02	3.34 ± 0.03	3.30 ± 0.02	3.29 ± 0.03	3.25 ± 0.02	3.24 ± 0.01	3.24 ± 0.01	3.22 ± 0.01
**MRS**	log CFU/ml	6.99 ± 0.03	7.10 ± 0.03	7.56 ± 0.02	8.35 ± 0.03	9.01 ± 0.04	9.40 ± 0.05	9.61 ± 0.04	9.70 ± 0.03	9.78 ± 0.05	9.84 ± 0.04	9.81 ± 0.04	9.84 ± 0.03	9.83 ± 0.08	9.71 ± 0.04	9.64 ± 0.05	9.15 ± 0.06	8.89 ± 0.05
	pH	5.71 ± 0.01	5.60 ± 0.02	5.55 ± 0.02	4.98 ± 0.03	4.44 ± 0.02	4.32 ± 0.02	4.18 ± 0.03	4.10 ± 0.02	4.02 ± 0.02	4.00 ± 0.01	4.01 ± 0.03	3.99 ± 0.02	3.98 ± 0.01	3.98 ± 0.02	3.97 ± 0.03	3.96 ± 0.04	3.96 ± 0.02

Each value was expressed as the mean of three independent experiments ± standard deviations analyzed in duplicate.

Table S3. Concentrations (mM) of organic acids (lactic and malic acids) and carbohydrates (glucose, fructose, sucrose, galactose and lactose) during the late exponential (LE) growth phase (16 or 18 h at 30°C) and the maintenance period (21 days at 4°C) in carrot (CJ) and pineapple (PJ) juices and MRS medium. Concentrations before fermentation are also reported.

	**CJ**			**PJ**			**MRS**		
	**Unfermented**	**LE growth phase**	**Maintenance**	**Unfermented**	**LE growth phase**	**Maintenance**	**Unfermented**	**LE growth phase**	**Maintenance**
Lactic acid (mM)	n.d.	41 ± 1^{d}	55 ± 2^{c}	n.d.	38 ± 2^{d}	57 ± 4^{c}	n.d.	128 ± 5^{b}	141 ± 8^{a}
Glucose (mM)	58 ± 2^{c}	50 ± 2^{d}	49 ± 2^{d}	206 ± 5^{a}	202 ± 4^{a}	198 ± 6^{a}	115 ± 4^{b}	53 ± 2^{d}	46 ± 3^{e}
Fructose (mM)	47 ± 1^{a}	42 ± 1^{b}	41 ± 2^{b}	212 ± 7^{a}	209 ± 3^{a}	207 ± 4^{a}	n.d.	n.d.	n.d.
Sucrose (mM)	56 ± 2^{a}	55 ± 2^{a}	55 ± 3^{a}	33 ± 2^{b}	29 ± 2^{bc}	26 ± 2^{c}	n.d.	n.d.	n.d.
Malic acid (mM)	29 ± 1^{a}	18 ± 1^{c}	13 ± 1^{d}	21 ± 1^{b}	14 ± 2^{d}	10 ± 1^{e}	n.d.	n.d.	n.d.
Citric acid (mM)	n.d.	n.d.	n.d.	20 ± 1^{a}	19 ± 1^{a}	19 ± 2^{a}	n.d.	n.d.	n.d.

Each value was expressed as the mean of three independent experiments ± standard deviations analyzed in duplicate.

Means within the row with different superscript letters (a-e) are significantly different ($P<0.05$).

n.d., not detected.

Table S4. Concentrations (mg l^{-1}) of total and individual free amino acids (FAA) during the late exponential (LE) growth phase (16 or 18 h at 30°C) and the maintenance period (21 days at 4°C) in carrot (CJ) and pineapple (PJ) juices and MRS medium. Concentrations before fermentation are also reported.

FAA (mg/L)	CJ			PJ			MRS broth		
	Unfermented	LE growth phase	Maintenance	Unfermented	LE growth phase	Maintenance	Unfermented	LE growth phase	Maintenance
Asp	228.12	151.12	241.23	83.01	54.02	221.38	155.31	58.71	88.45
Thr	28.72	16.13	23.12	11.13	9.41	26.41	141.43	123.22	202.33
Ser	212.72	161.13	211.90	88.20	88.23	203.36	209.22	38.32	61.71
Glu	50.59	22.03	17.21	57.30	27.12	71.55	446.18	399.47	559.23
Gly	7.61	1.12	1.81	17.21	15.61	34.62	270.56	463.51	620.34
Ala	172.13	123.61	173.25	55.72	31.74	92.38	368.12	367.63	570.74
Cys	8.90	6.96	8.23	12.30	12.21	34.53	100.72	102.31	163.66
Val	40.23	21. 12	29.15	20.27	22.24	53.12	196.19	210.50	327.38
Met	6.13	2.46	2.49	18.21	11.12	28.41	139.72	122.24	178.41
Ile	23.63	7.31	13.02	13.71	12.22	27.66	198.61	136.31	219.69
Leu	16.12	5.13	6.73	11.36	16.74	39.02	390.80	323.12	527.15
Tyr	15.12	3.21	5.03	19.45	13.22	35.51	94.36	22.23	40.12
Phe	26.13	18.13	22.13	33.62	26.15	61.32	273.61	182.41	308.36
His	9.12	6.12	7.03	41.71	32.41	60.12	184.60	131.36	225.81
Trp	7.21	4.20	6.02	14.14	11.12	12.01	20.22	81.57	47.38
Orn	1.32	2.03	2.31	2.41	1.41	3.23	12.56	13.73	28.50
Lys	9.12	17.03	25.01	20.30	14.85	44.10	180.38	141.80	222.03
Arg	35.21	20.03	36.96	16.71	17.20	45.68	299.12	282.39	436.43
Pro	13.73	11.02	16.92	11.23	13.81	24.49	84.71	120.91	124.67
GABA	183.12	129.20	172.71	26.13	27.51	58.21	33.52	83.20	107.25
Total	1095 ± 37^{e}	708 ± 63^{f}	1022 ± 81^{e}	574 ± 37^{g}	458 ± 37^{h}	1177 ± 43^{d}	3800 ± 61^{b}	3405 ± 72^{c}	5060 ± 86^{a}

Each value was expressed as the mean of three independent experiments ± standard deviations analyzed in duplicate.

Means within the row with different superscript letters (a-h) are significantly different (*P*<0.05).

Table S5. Comparison of microarray and qRT-PCR data for differentially expressed genes in *Lactobacillus plantarum* C2 during the late exponential (LE) growth phase (16 or 18 h at 30°C) in carrot (CJ) or pineapple (PJ) juices compared to MRS medium.

	**Carrot juice (CJ)**	
**Gene**	**Microarray fold change**	**qRT-PCR fold change**
lp_3491	7.67	7.62
hpk5	6.20	2.99
lp_2183	3.49	1.94
lp_1425	6.59	1.7
gadB	-11.28	0.76
purF	-3,29	0.77
pyrC	-7.00	0.63
pyrB	-8.62	0.34
aroC2	12.30	19.95
aroI	2.14	10.66
	**Pineapple juice (PJ)**	
**Gene**	**Microarray fold change**	**qRT-PCR fold change**
fabI	2.92	3.08
fabD	2.66	4.64
fabz1	3.23	4.99
hisX	2.11	1.48
hisD	2.04	2.2
atpG	2.07	1.71
atpA	2.12	3.47
pdhC	-7.43	0.13
accA2	2.74	1.75
accD2	2.41	5.64
aroC2	9.83	15.63
aroI	3.43	12.01
aroF	4.59	2.42
oppA	3.81	12.40
oppD	4.49	3.17
ileT	2.47	1.86
bcap	3.66	3.27
lp_1425	4.52	1.80
lp_3491	9.10	11.59
dltX	4.84	1.64
pyrB	-2.05	0.53

Table S6. Primer sets used for qRT-PCR.

**Target gene**		**Sequence 5'→3'**
16S rRNA	Forward primer	GCGGTAATACGTAGGTGG
	Reverse primer	TCCTCTTCTGCACTCAAGT
recA	Forward primer	ACGGCGGGCAGAACAGATCAA
	Reverse primer	TCGGCACGCTTAAATGGCGGT
accA2	Forward primer	CGGGTGCCTATCCGGGTGTT
	Reverse primer	TGCCACCTTCACCAACAATCAAACT
accD2	Forward primer	TATGGGTTTCGCTTGCCTGCTTG
	Reverse primer	ACTGACGGGCGCACTTAGGTC
dltX	Forward primer	CCAAGGCGAGCATGACAGCAAAG
	Reverse primer	AGTGGAGGAGGTGATGGCATGTT
fabI	Forward primer	TCAAAACGTTGGCGGTAACGGG
	Reverse primer	CAGCCACGTTGCCGATCTCAC
fabD	Forward primer	AGCAGCGAGTGAGGCGTTGG
	Reverse primer	CCTGGCTAAAGGGGGTCACGG
hisD	Forward primer	TCCTAAGAAGACCGACCCGGCA
	Reverse primer	ACGGTGGCACCAGAACACTTGG
hisX	Forward primer	GGCCGTAAGACCAAGGTGCGA
	Reverse primer	TCGCGCCAATTGCAACGCC
atpG	Forward primer	TCGCGGTTGACGACGAAGCA
	Reverse primer	GTCGGCCGTGTTGCCACAAT
atpA	Forward primer	CCGCCACCCAATTCATCGCTC
	Reverse primer	CGTCGTCCTCCAGGTCGTGAA
pdhC	Forward primer	TTGGGCCGTTGCACCATCAATC
	Reverse primer	GGTGTTGGGCGTATCGGTAAGGAA
aroI	Forward primer	ACATGCAACACATTCGTGGTAAGGA
	Reverse primer	GGTTCCGCAATCTGCATCCCA
aroF	Forward primer	CGCTCTACGCTTGCATTTTGGCT
	Reverse primer	AAGGCCGCCATGAAGCCGAT
aroC2	Forward primer	AGACCGCTTCGACCGCTAACT
	Reverse primer	TCGTGAGGTCAGAGTGATGATTGGG
oppA	Forward primer	TCGTCAATGGCAAGAAGCCCGT
	Reverse primer	GCCCATCAGCTTGTCAAAGTAAGCA
oppD	Forward primer	ACATGCAACACATTCGTGGTAAGGA
	Reverse primer	GGTTCCGCAATCTGCATCCCA
Bcap	Forward primer	AACGGTCGCAACGGCTAGGA
	Reverse primer	TCGCCGGTTCCGCGTATTCA
ileT	Forward primer	GCCCGAGCTGGCTTCTTCTCA
	Reverse primer	AAAGGCGACCCCGCCGTTATC
lp_1425	Forward primer	GTGGTCAACGGACTGATTTGAACGG
	Reverse primer	CCACTTGACTGAATCCAGGGCGT
lp_3491	Forward primer	GGCACTCTTCAAGGCAGCTGGAAT
	Reverse primer	GGATCGGGAACCGGCAAGTGT
fabz1	Forward primer	GGGTGAATCGATCGTGGTGACGAAA
	Reverse primer	CGGCTTGCGCCAAAGCTTCAAT
pyrB	Forward primer	GTCACGGCCATACCATTCTTTGGGG
	Reverse primer	CCATTCACGTGTGGCCCGTTC
pyrC	Forward primer	TGCCATAAACGGGCCAACCGA
	Reverse primer	TGGGGGCACCGGCTGATCTA
purF	Forward primer	CGATCCGCCGACTGGTGGTA
	Reverse primer	TTGCGAGAACAGGGGGTTCGG
hpk5	Forward primer	AGTGATGCTGAATTAGTTGGGCGGG
	Reverse primer	CGCCAAATTATCAGTGACCTGGGTG
lp_2183	Forward primer	GGGTGACGTGTAGAATTGGGCGA
	Reverse primer	GTGAGACCATGGACCAGGCGG
gadB	Forward primer	AGCCGCACTCGATAAGGTCGT
	Reverse primer	AGCCAACCGGAAGTCCCAGA
